# Synergy of Ginkgetin and Resveratrol in Suppressing VEGF-Induced Angiogenesis: A Therapy in Treating Colorectal Cancer

**DOI:** 10.3390/cancers11121828

**Published:** 2019-11-20

**Authors:** Wei-Hui Hu, Gallant Kar-Lun Chan, Ran Duan, Huai-You Wang, Xiang-Peng Kong, Tina Ting-Xia Dong, Karl Wah-Keung Tsim

**Affiliations:** 1 Shenzhen Key Laboratory of Edible and Medicinal Bioresources, HKUST Shenzhen Research Institute, Hi-Tech Park, Nanshan, Shenzhen 518057, China; whuaf@connect.ust.hk (W.-H.H.); gallant@ust.hk (G.K.-L.C.); duanran@ust.hk (R.D.); hyw@ust.hk (H.-Y.W.); xpkong@ust.hk (X.-P.K.); botina@ust.hk (T.T.-X.D.); 2 Division of Life Science and Center for Chinese Medicine, The Hong Kong University of Science and Technology, Clear Water Bay Road, Hong Kong 999077, China

**Keywords:** ginkgetin, resveratrol, synergism, angiogenesis, VEGF, colon cancer

## Abstract

Ginkgetin, a biflavone from *Ginkgo biloba* leaf, and resveratrol, a polyphenol found in grape and wine, are two phytochemicals being identified for its binding to vascular endothelial growth factor (VEGF): the binding, therefore, resulted in the alteration of the physiological roles of VEGF-mediated angiogenesis. The bindings of ginkgetin and resveratrol were proposed on different sites of VEGF, but both of them suppressed the angiogenic properties of VEGF. The suppressive activities of ginkgetin and resveratrol in VEGF-mediated angiogenesis were supported by several lines of evidence including (i) inhibiting the formation of sub-intestinal vessel in zebrafish embryos and microvascular sprouting in rat aortic ring; and (ii) suppressing the phosphorylations of VEGFR2, Akt, eNOS, and Erk as well as expressions of matrix metalloproteinases (MMPs), MMP-2, and MMP-9 in human umbilical vein endothelial cells (HUVECs). Here, we showed the synergy of ginkgetin and resveratrol in suppressing the VEGF-induced endothelial cell proliferation, migration, invasion, and tube formation. The synergy of ginkgetin and resveratrol was further illustrated in HT-29 colon cancer xenograft nude mice. Ginkgetin and resveratrol, when applied together, exerted a synergistic anti-tumor effect of 5-fluorouracil with decreasing microvessel density of tumors. In parallel, the combination of ginkgetin and resveratrol synergistically relieved the 5-fluorouracil-induced inflammatory response by suppressing expressions of COX-2 and inflammatory cytokines. Thus, the anti-angiogenic roles of ginkgetin and/or resveratrol could provide effective therapeutic strategy in cancer, similar to that of Avastin, in suppressing the VEGF-mediated angiogenesis during cancer development.

## 1. Introduction

Angiogenesis is formation of neovessels from pre-existing vessels, and has been proven to play significant roles in both normal and pathological conditions, particularly in cancer development [1]. This process involves complex interactions among endothelial cells, angiogenic factors, and extracellular matrix components [2]. Angiogenesis can be grouped into several responses, for example, endothelial proliferation, migration, and differentiation, and protease induction. There are different angiogenesis-related growth factors in regulating the synchronization of complex events of angiogenesis. Among these activators, compelling evidence has suggested that vascular endothelial growth factor (VEGF) is the most potent angiogenic stimulator [3,4]. There are five members in the VEGF family: VEGF-A, -B, -C, -D, and placental growth factor (PlGF) [5]. Among them, VEGF-A was the first one to be discovered and is the most investigated member, usually regarded as VEGF [6].

The specific effect of VEGF on endothelial cells is primarily regulated by two types of receptor tyrosine kinases, namely vascular endothelial growth factor receptor-1 (VEGFR-1) and VEGFR-2. Blocking the signaling of VEGF-mediated responses is a popular target in suppressing cancer growth. Several therapeutic drugs against receptor tyrosine kinases in oncology have been developed. They can be approximately divided into three categories: antibodies targeting VEGF, antibodies targeting VEGFR-2, and small molecular inhibitors targeting VEGFR-2 kinase domain [7]. Bevacizumab (Avastin), a recombinant humanized monoclonal antibody from Genetech [8], has been approved by the Food and Drug Administration (FDA) for the treatment of non-small-cell lung cancer, metastatic colorectal cancer, and glioblastoma [9]. VEGF Trap (aflibercept), a novel soluble decoy receptor from Regeneron Pharmaceuticals, employs the fusion of components from endogenous receptors [10]. Thus, the discovery of inhibitors blocking the VEGF/VEGFR2 signaling pathway could be a promising new strategy in treating angiogenesis-related diseases.

Colorectal cancer (CRC), one of the most commonly registered cancers, has a high mortality rate, especially for advanced and metastatic patients [11]. The most commonly-used treatments for CRC include radiotherapy, chemotherapy, and surgery. Chemotherapy is the primary method that is always implemented in cancer treatment. Unfortunately, chemotherapy not only destroys malignant cells, but also damages normal tissues and has dangerous side effects. Although surgery can cure ~50% of CRC patients, the disease recurrence is ~50% among patients with CRC receiving resection [12]. 5-Fluorouracil (5-FU) is one of the most commonly-used and inexpensive chemotherapeutic agents for CRC treatment [13,14], which irreversibly suppresses the synthase of thymidylate, leading to a shortage of DNA synthesis [15]. The efficacy of 5-FU has limitations due to its toxicity and drug resistance. They are also not specific to cancer cells. Thus, combining 5-FU with other agents provides a more effective approach to make cancer cells sensitized to chemotherapy while reducing the toxicity to normal cells. Abnormal angiogenesis is a critical step in CRC progression by providing oxygen and nutrients for the survival, growth, and metastasis of tumor cells [16]. Thus, a combination of anti-angiogenic therapy and chemotherapy, as a first-line treatment for metastatic CRC, has been established [17].

Ginkgetin is a biflavone mainly isolated from *Ginkgo biloba* leaves: the extract is a health food supplement sold commonly in the market, and contains ~0.65% of ginkgetin [18]. The leaf extract of *Ginkgo biloba* has been demonstrated to reduce HIF-1α and VEGF expression in retinal pigment epithelial cells [19]. Ginkgetin has been shown to exhibit a series of pharmaceutical activities including anti-inflammatory, neuroprotective, anti-fungal, and anti-tumor activities [20]. Resveratrol, a polyphenol found in grape and wine, was shown to exert suppressive effects on endothelial cell migration, cell invasion, and tube formation as well as to attenuate the formation of neo-vessels in zebra fish embryo in vivo [21]. Both ginkgetin and resveratrol are widely consumed in health food supplements, and are known to have low toxicity in humans [22,23]. Here, we provided several lines of evidence to show the binding of ginkgetin and resveratrol onto VEGF, and therefore the pharmaceutical activities with the combination of ginkgetin and resveratrol in angiogenesis during cancer development were elucidated. By using in vitro and in vivo models, we analyzed the effects of ginkgetin–resveratrol in the anti-tumor activity of 5-FU in mice bearing human (HT-29) colon cancer. The combination of ginkgetin and resveratrol synergized the chemotherapeutic effect of 5-FU in colon cancer through anti-angiogenesis modulation. 

## 2. Results

### 2.1. Ginkgetin Binds Vascular Endothelial Growth Factor and Regulates Angiogenesis

**Figure 1:**
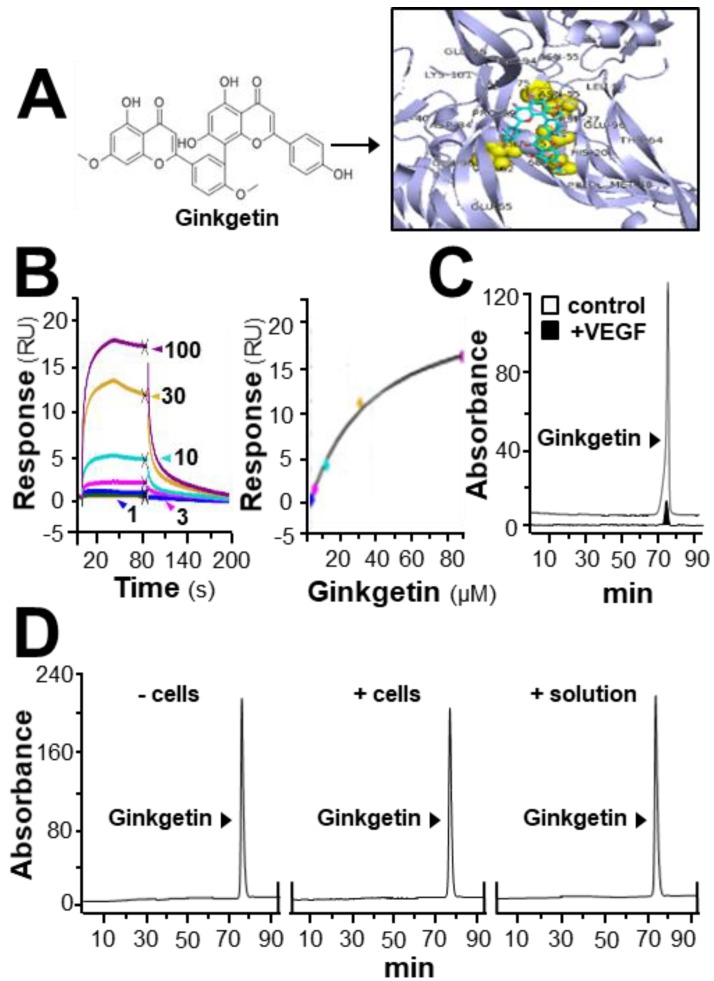
Ginkgetin binds with VEGF. (**A**) The structure of ginkgetin (**left**) was directly downloaded from the NCBI-PubChem database and the VEGF structure was obtained from Protein Data Bank for molecular docking with AutoDock software. Visualization of the binding interaction between ginkgetin and VEGF was demonstrated (right). VEGF: blue; resveratrol: sticks; carbon color: cyan-blue; oxygen: red; hydrogen: silver; the predicable binding site: yellow. (**B**) In the Biacore assay, a series of different concentrations of ginkgetin (from 1 to 100 μM, as indicated; left) were performed to flow through the surface of the chip from 10 s to 80 s, and then, after 80 s, running buffer was used to flow through the surface. The dose-response curve is shown (right). (**C**) Ultra performance liquid chromatography chromatogram was to detect ginkgetin in the supernatant after biotinylated VEGF or VEGF (66.1 ng/mL) in an immunoprecipitation assay by streptavidin magnetic beads. (**D**) High performance liquid chromatography chromatogram was used to detect the level of applied ginkgetin (1 μM) in the treated cells for 24 h. “+/− cells” show that ginketin was incubated with or without cells. “+ solution” represents that ginketin was incubated with water only. After incubation, the amount of input ginkgetin is shown. Representative figure is shown, *n* = 5.

In HerboChips drug screening, the biotinylated VEGF showed positive signals in our previous screening to the extracts of *G. biloba* leaves (Supplementary Figure S1). By using the PyMOL molecular graphics system and AutoDock tools, molecular docking was performed. The interaction between ginkgetin and VEGF protein was demonstrated (Figure 1A), of which the binding affinity was analyzed by using vina software. The binding affinity between VEGF and ginkgetin was proposed to be −8.5 to −7.7. The auto-docking result proposed that the binding site of resveratrol to the VEGF protein could lie at the interacting domain between VEGF and its receptor (Figure 1A). The binding of ginkgetin to VEGF could be further illustrated by surface plasmon resonance. Based on the results obtained from a Biacore machine, an increasing response value showing ginkgetin–VEGF interaction was shown in a concentration-dependent manner from 1 to 100 µM (Figure 1B). Moreover, the interaction between ginkgetin and VEGF in vitro was further confirmed in an immuno-precipitation assay. When compared with the control, the amount of ginkgetin in the supernatant with biotinylated VEGF treatment was much lower (Figure 1C). The aforementioned results, therefore, support direct binding between VEGF and ginkgetin. Furthermore, the stability of ginkgetin was further confirmed by a high performance liquid chromatography method. The amount of applied ginkgetin was unchanged during the drug treatment in culture (Figure 1D).

**Figure 2:**
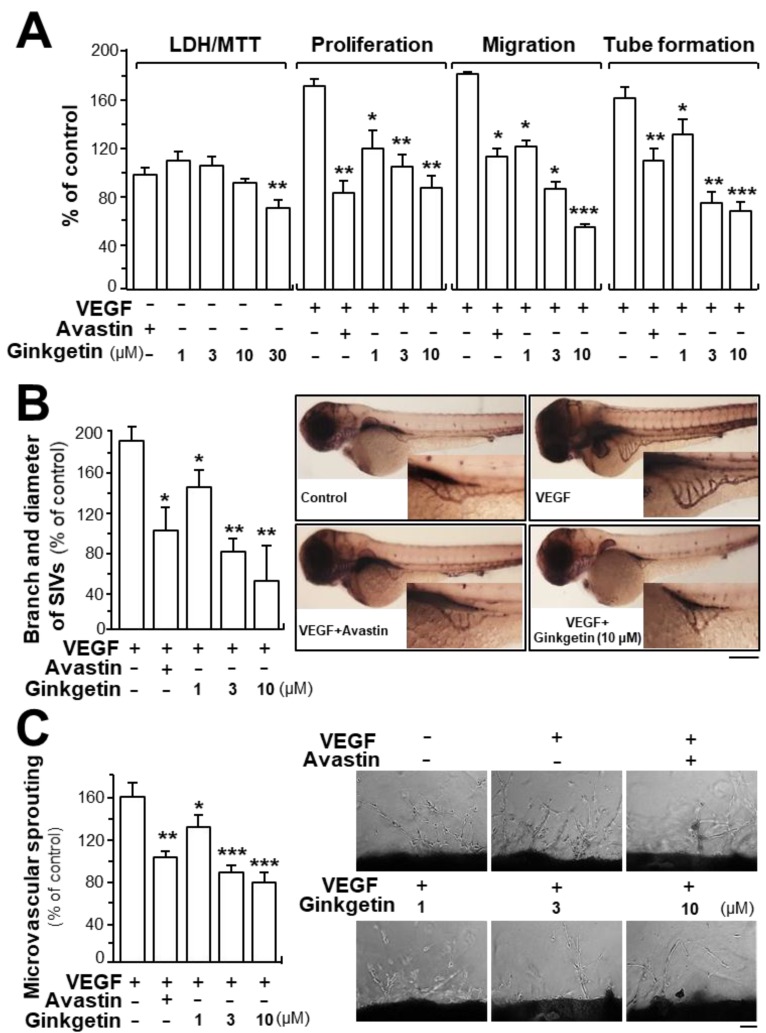
Ginkgetin inhibits angiogenesis. (**A**) Ginkgetin was applied onto human umbilical vein endothelial cells (HUVECs) for 48 h, and 3-(4,5-dimethylthiazol-2-yl)-2,5-diphenyltetrazolium bromide (MTT) assay and lactate dehydrogenase (LDH) cytotoxicity assay were determined. In cell migration, a total of 20 × 10^4^ HUVECs were placed onto a 12-well plate. An artificial wound was manually created by scratching the cell monolayer at the bottom of wells, and the images of wounds were photographed at 0 h and 8 h separately under a phase-contrast microscope. In tube formation, cultures were as above. Photos of endothelial cell tube-like structures were taken under a phase-contrast microscope. Here, VEGF (10 ng/mL) and Avastin (200 μg/mL) were used in all cases. (**B**) Healthy zebrafish embryos were incubated with phenylthiourea water containing VEGF (10 ng/mL), ginkgetin, or Avastin on the first day of development. After treatment for 48 h, the embryos were stained. The area and branches of sub-intestinal vessels that exerted effects by a series of various concentrations of drug were quantified. (**C)** Thoracic aortas were removed from 6-week-old rats, and aortic ring fragments (cut 1-mm thick) were cultured in Matrigel and incubated with VEGF with or without ginkgetin or Avastin for eight days. Images represent the microvascular outgrowth, and the area of microvascular sprouting in the control group and drug-treated group was quantified with ImageJ software. Results are shown as the percentage of change when compared to the control (no drug) in terms of mean ± SEM, where *n* = 3–4; * *p* < 0.05; ** *p* < 0.01; *** *p* < 0.001 vs. the VEGF-treated group.

Due to the binding of ginkgetin with VEGF, we predicted that ginkgetin could contribute to the VEGF-mediated angiogenesis and endothelial cell proliferation. Treatment of ginkgetin on human umbilical vein endothelial cells exerted no effect on cell viability with concentrations up to 10 µM or any effects on cell proliferation, migration, and tube formation (Figure 2A). In contrast, the VEGF-induced endothelial cell proliferation was significantly blocked by the application of ginkgetin, in a concentration-dependent manner (Figure 2A). Cell migration and tube formation have vital importance in cell growth and differentiation of endothelial cells. Here, the endothelial cell migration was potentiated after VEGF treatment: ginkgetin apparently inhibited the VEGF-mediated cell migration, or wound recovery, in a concentration-dependent manner (Figure 2A). After VEGF treatment, elongated and solid capillary-like tubes were obviously demonstrated, while interruptions in capillary-like tubes were apparently revealed after different concentrations of ginkgetin were applied (Figure 2A). Avastin, exerting inhibitory effects on angiogenesis, served as a positive control.

Neo-vascularization, located in the zebrafish sub-intestinal vessel, was monitored in the presence of ginkgetin. After dechlorination, the fish embryos were fed with VEGF, Avastin, or a series of different concentrations of ginkgetin. Apparent and elongated vessels in the sub-intestinal vessel were triggered after VEGF treatment, and moreover, the area was broadened (Figure 2B). After exposure to ginkgetin or Avastin, the area located in the sub-intestinal vessels was smaller as well as having less branches (Figure 2B). These results support the conclusion that ginkgetin exerts an inhibitory effect on VEGF-triggered angiogenesis of endothelial cells in vivo.

The inhibitory effect of ginkgetin on VEGF-mediated angiogenesis was also investigated by performing an ex vivo aortic ring sprouting assay. Rat aortic fragments were placed onto Matrigel and incubated with VEGF, Avastin, or ginkgetin. The microvascular sprouting, located at the luminal cut edges of the aortic section, was clearly developed with applied VEGF while fewer micro-vessels were formed with treatment of Avastin (Figure 2C). In the present of the co-applied VEGF, ginkgetin significantly inhibited the VEGF-mediated neovascularization in aortic fragments (i.e., decreased the microvascular outgrowth) in a concentration-dependent manner (Figure 2C).

**Figure 3:**
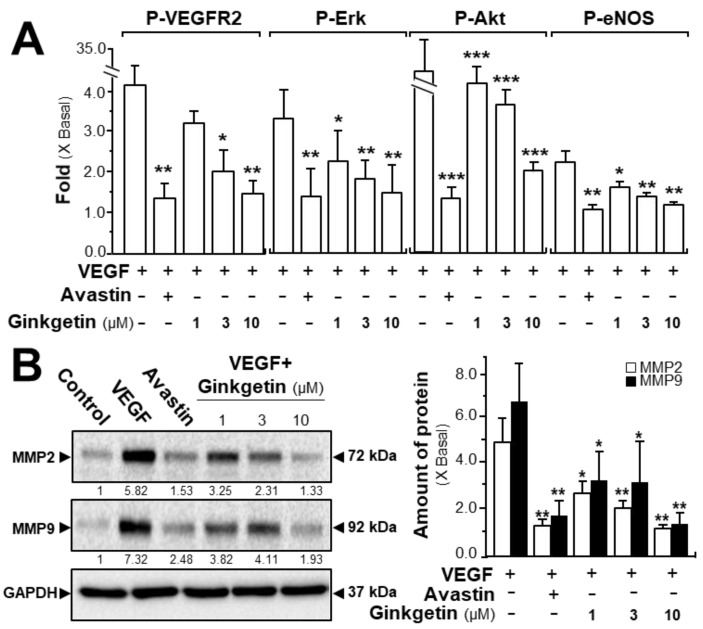
Ginkgetin blocks the VEGF-mediated signaling mechanism. (**A**) HUVECs were plated into each well of a 12-well plate as in Figure 2. Cell lysates were collected after 10 min of treatment. Phosphorylated and total protein expressions of VEGFR2 at ~210 kDa and ~230 kDa, Erk at ~42 kDa and ~44 kDa, Akt at ~60 kDa, and eNOS at ~140 kDa were detected by western blotting. Avastin (200 μg/mL) served as a control. Quantitation of VEGFR2/Erk/Akt/eNOS phosphorylation was shown in a dose-dependent manner at 10 min. (**B**) HUVECs, at a density of 40 × 10^4^ per well, were seeded in a 6-well plate. VEGF (10 ng/mL) was applied with or without Avastin or ginkgetin for 48 h. Cell lysates were collected after drug treatment. MMP-2 (~72 kDa), MMP-9 (~92 kDa), and GAPDH (~37 kDa, control) were determined (left). Quantitation was done from the band intensity in western blotting (right). Results are expressed as the fold of change compared to the control (X Basal), where the control was set as 1, mean ± SEM, where *n* = 4–5; * *p* < 0.05; ** *p* < 0.01; *** *p* < 0.001 vs. VEGF-treated group.

The phosphorylations of VEGFR, Erk, Akt, and eNOS were determined here. After activation of VEGF in HUVECs, the tyrosine phosphorylation of VEGFR2, an active form of VEGFR2, was identified. Western blotting was used to determine the protein expression of phosphorylated VEGFR2 (Tyr 1175) and total VEGFR2 (Supplementary Figure S2). The application of VEGF significantly increased the phosphorylation/activation of VEGFR2, which was shown in a time-dependent manner, and the maximal activation was at ~5-fold after 10 min of VEGF induction (Figure 3A). Meanwhile, ginkgetin application in HUVECs markedly inhibited the VEGF-induced VEGFR2 phosphorylation in time- and dose-dependent manners with the amount of VEGFR2 protein unaltered (Figure 3A and Supplementary Figure S2). The downstream signaling, mediated by VEGFR2, was further investigated in a scenario of ginkgetin-treated HUVEC cultures (Supplementary Figure S2). In endothelial cells, the application of VEGF potentiated the phosphorylations of Erk, Akt, and eNOS by ~2- to ~30-fold with the protein amounts of Erk, Akt, and eNOS unchanged (Figure 3A). The treatment with ginkgetin could apparently block the VEGF-mediated protein expressions of phosphorylated Erk, Akt, and eNOS in time- and concentration-dependent manners, and Avastin showed similar inhibitory effects on the VEGF-induced phosphorylations of the targeted molecules (Figure 3A and Supplementary Figure S2).

We further evaluated the signaling mechanism of ginkgetin in angiogenesis by measuring the expression of the factors closely related with cell migration and invasion [24]. The expression levels of MMP-2 and MMP-9 were determined by western blotting, with or without ginkgetin treatment, in VEGF-treated endothelial cells. VEGF application markedly increased the expressions of MMP-2 and MMP-9 by ~5-fold and ~6-fold, respectively (Figure 3B). Meanwhile, Avastin suppressed the VEGF-induced expressions. In cultured HUVECs, the treatment of ginkgetin inhibited VEGF-mediated expressions of MMP-2 and MMP-9 proteins at ~2-fold and ~4-fold, respectively (Figure 3B). On the other hand, resveratrol, another popular phytochemical, has been previously shown to suppress VEGF-mediated angiogenesis, similar to that of ginkgetin as described here; however, the binding site was proposed to be different to that of ginkgetin [21].

### 2.2. Synergy of Ginkgetin and Resveratrol in VEGF-Mediated Angiogenesis

**Figure 4:**
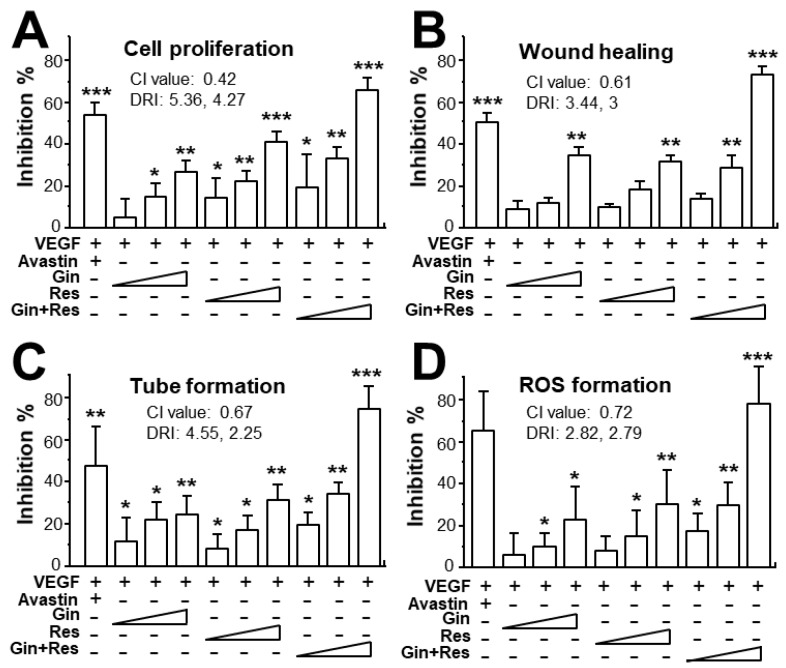
Synergy of ginkgetin and resveratrol inhibits VEGF-mediated angiogenesis. (**A**) In cell proliferation assay, the endothelial cells were plated in a 96-well plate at a density of 5,000 cells/well, and incubated in the presence or absence of drugs for 48 h. (**B**) HUVECs, at a density of 20 × 10^4^ cells/well), were seeded onto each well of a 12-well plate. A wound was created by scratching the cell monolayer manually, and images of wounds were separately taken separately at 0 h and 8 h with application of a phase-contrast microscope. (**C**) In tube formation assay, HUVECs were seeded onto a 12-well plate with the cell density set at 20 × 10^4^ per well. Each well was pre-coated with matrigel. VEGF, with or without different concentrations of drugs, was applied for 8 h. To quantify the pictures of tube formation, three fields in one photo were randomly determined and branching points were recognized manually. (**D**) For ROS formation, a total of 20 × 10^4^ HUVECs in each well of a 12-well plate were treated with 2’,7’–dichlorofluorescin diacetate (DCF-DA) at 37 °C for 30 min before other treatment for 48 h. The level of intracellular ROS was tested with the application of laser confocal fluorescent microscopy. In all cases, VEGF (10 ng/mL) and Avastin (200 μg/mL) were used here. In the ginkgetin-treated group, the working concentrations were 0.1, 0.3, and 1 μM. In the resveratrol-treated group, the working concentrations were 0.3, 1, and 3 μM. In the combined ginkgetin and resveratrol-treated group, the low working concentrations were 0.1 μM for ginkgetin and 0.3 μM for resveratrol; the middle working concentrations were 0.3 μM for ginkgetin and 1 μM for resveratrol; the high working concentrations were 1 μM for ginkgetin and 3 μM for resveratrol. The CI and DRI values are shown. Data are expressed as the mean ±SEM of the percentage of change when compared to VEGF-treated group, where *n* = 3–5; * *p* < 0.05; ** *p* < 0.01; *** *p* < 0.001 vs. the VEGF-treated group.

**Figure 5:**
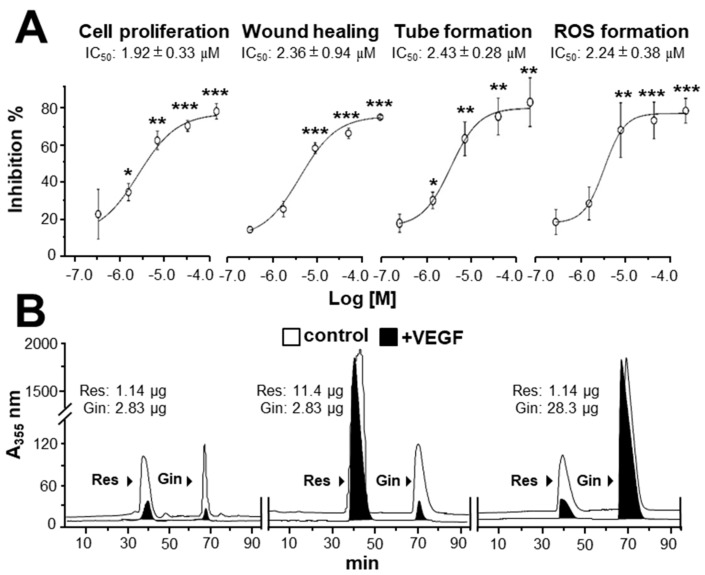
Binding of ginkgetin and resveratrol onto VEGF suppresses angiogenesis. (**A**) The assay and treatment of HUVECs were similar to that in Figure 4 for 48 h. VEGF (10 ng/mL) and Avastin (200 μg/mL) were used. A stock solution of combined ginkgetin–resveratrol was made by equally mixed ginkgetin (20 mM) and resveratrol (60 mM), and the left concentrations were made from a stock solution separately diluted by 3×, 10×, 30×, and 100×. For cell treatment, all stock solutions were diluted 1000× by the medium. The highest working concentrations were 10 μM for ginkgetin and 30 μM for resveratrol; the lowest working concentrations were 0.1 μM for ginkgetin and 0.3 μM for resveratrol. The IC_50_ values are shown for each assay. Data are expressed as the mean ±SEM of the percentage of change when compared with the VEGF-treated group, where *n* = 4; * *p* < 0.05; ** *p* < 0.01; *** *p* < 0.001 vs. the VEGF-treated group. (**B**) An ultra-performance liquid chromatography chromatogram was used to detect the amount of resveratrol and ginkgetin in the supernatant after biotinylated VEGF or VEGF (66.1 ng/mL) in an immunoprecipitation assay by streptavidin magnetic beads. The amount of input phytochemicals are shown. A typical figure is demonstrated, where *n* = 5.

Due to the possible different binding sites of ginkgetin and resveratrol with VEGF, we tested the synergistic inhibition of combined ginkgetin and resveratrol in VEGF-mediated angiogenesis. To further evaluate the synergistic inhibitory effects, various doses of ginkgetin and resveratrol were mixed together at a 1:3 ratio: this combination ratio has been shown to have better synergy. The inhibition on VEGF-mediated angiogenic activities were analyzed including endothelial cell proliferation, cell migration, tube formation, and ROS formation (Figure 4). As shown in Figure 4A, the inhibition of combined ginkgetin and resveratrol on cell proliferation was increased when compared with that of a single drug alone. To analyze the relationship of drug-to-drug interaction, the median-effect principle was employed to evaluate the possibility of synergism. With the application of the classical isobologram equation of Chou–Talalay, a combination index (CI) was determined. For the cell proliferation assay, when Fa = 0.5, the calculated CI value was 0.42, suggesting that the inhibitory effect of the ginkgetin and resveratrol combination might be synergistical (Figure 4A). Furthermore, the drug reducing index (DRI) values were 2.98 and 2.66, suggesting that a dose reduction might be applied in therapeutic application. In parallel, for the wound healing, tube formation, and ROS formation assay (Figure 4B–D), when Fa = 0.5, the calculated CI values were 0.61, 0.67, 0.72, respectively, again suggesting that their synergy. In addition, the DRI values were all greater than 1. The inhibition curves were calibrated. The combination of ginkgetin and resveratrol exerted inhibitory effects on these VEGF-mediated angiogenic functions in a dose-dependent manner (Figure 5A). The IC_50_ values for these VEGF-induced activities were also determined, as shown.

By using the PyMOL system, the binding site of ginkgetin with VEGF was different from the interactions of resveratrol and VEGF [20]. To confirm the possible binding site between ginkgetin and resveratrol to VEGF, an immuno-precipitation assay was applied. When compared with the control, the amounts of ginkgetin and resveratrol in the supernatant with biotinylated VEGF treatment were reduced (i.e., precipitated) (Figure 5B). Furthermore, the binding of ginkgetin to VEGF (i.e., precipitated) in the supernatant was not affected in an excessive increasing amount of resveratrol (Figure 5B). In parallel, the overloaded amount of ginkgetin exerted no effect on the binding between resveratrol and VEGF (Figure 5B). The aforementioned results support the conclusion that the binding site of ginkgetin and VEGF was different from that of resveratrol.

### 2.3. Ginkgetin and Resveratrol Synergy in Enhancing 5-Fluorouracil in Colon Cancer Xenograft Mice

**Figure 6:**
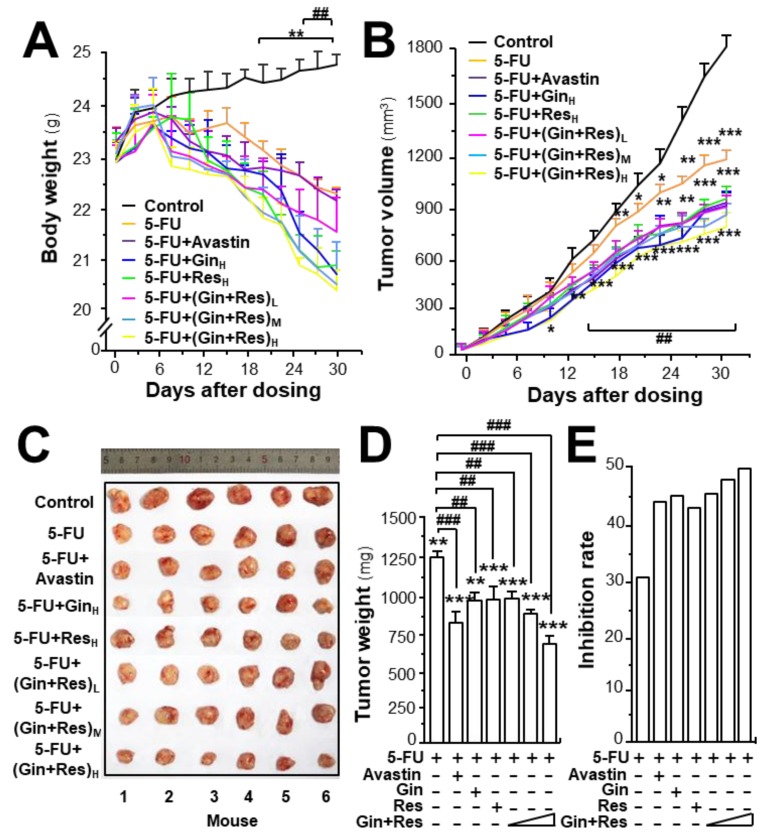
Synergy of ginkgetin and resveratrol suppresses tumor growth in nude mice. Xenograft nude mice were established by implanting HT29 colon cancer cells subcutaneously in the right flank of mice. The tumors were allowed to grow at ~90 mm^3^. Thereafter, the 5-FU (30 mg/kg/2 days, i.p.) Avastin group (6 mg/kg/2 days, i.p.) ginkgetin group (800 mg/kg/day, i.g.), resveratrol group (1920 mg/kg/day, i.g.), ginkgetin and resveratrol low-dose group (80 mg/kg/day + 240 mg/kg/day, i.g.), ginkgetin and resveratrol middle-dose group (160 mg/kg/day + 480 mg/kg/day, i.g.), and ginkgetin and resveratrol high-dose group (320 mg/kg/day + 960 mg/kg/day, i.g.) were administered. Mice in each group were administered 5-FU, except the control group. (**A**) The body weight was measured. (**B**) The mean tumor volume (in cm^3^) was calculated using the ellipsoid formula: (D × (d2))/2, where “D” represents the large diameter of the tumor, and “d” represents the small diameter. (**C**) Mice bearing tumors were sacrificed at day 30, and the tumors are shown. (**D**) Mean tumor weight at the end of treatment. (**E**) Inhibitory rates of drug-treated groups. The tumor inhibitory rate was calculated as follows: IR (%) = (1 – TWt/TWc) × 100, where TWt and TWc are the mean tumor weight of the drug treated and control groups, respectively. “5-FU + Gin_H_” refers to the treatment of 5-FU and ginkgetin at high dosage. “5-FU + Res_H_” refers to the treatment of 5-FU and resveratrol at high dosage. “5-FU + (Gin + Res)_L_” refers to the treatment of 5-FU and combined ginkgetin–resveratrol at low dosage. “5-FU + (Gin + Res)_M_” referred to the treatment of 5-FU and combined ginkgetin–resveratrol at middle dose. “5-FU + (Gin + Res)_H_” refers to the treatment of 5-FU and combined ginkgetin–resveratrol at high dose. Data are expressed as mean ± SEM of the percentage of change when compared with the control, where *n* = 8; *) *p* < 0.05; ** *p* < 0.0; *** *p* < 0.001 vs. the control group; ## *p* < 0.01; ### *p* < 0.001 vs. the 5-FU group.

To investigate the in vivo anti-angiogenesis effect of combined ginkgetin and resveratrol, an HT-29 colon cancer xenograft was developed. The efficacy of combined phytochemicals together with 5-FU on colon tumor growth was evaluated. Avastin, exerting an enhanced chemotherapeutic efficacy of 5-FU, served as a positive control [25]. The body weight of tumor-bearing mice was decreased after 5-FU treatment, and the weight reduction was restored in 5-FU-treated mice by application of Avastin, ginkgetin, resveratrol, or ginkgetin + resveratrol (Supplementary Figures S3A and S5A, Figure 6A). In control mice, the tumor volume gradually increased in a time-dependent manner (Supplementary Figures S3B and S5B, Figure 6B). Treatment of 5-FU significantly suppressed the tumor volume, and the reduction was more robust when co-treated 5-FU with Avastin or the phytochemicals. The highest suppression of tumor growth was observed in mice receiving 5-FU and the ginkgetin–resveratrol combined treatment, which was better than that of Avastin, or a single application of ginkgetin–resveratrol (Supplementary Figures S3B, S5B, and Figure 6B). After 30 days of treatment, the tumors were removed and weighed. Similarly, a significant difference was observed in tumor weights of the control and 5-FU-treated mice, and again, a synergistic effect was observed in 5-FU and the phytochemically co-treated mice (Supplementary Figures S3C and S5C, Figure 6C). Compared to the 5-FU-treated tumor-bearing mice, the co-treatment of ginkgetin–resveratrol with 5-FU reduced the tumor weight by >50%. The co-treatment of 5-FU and Avastin showed similar suppressive effects on tumor weight (Supplementary Figures S3D and S5D, Figure 6D). The tumor inhibitory rate was further compared in different treatments. The inhibitory rate was calculated as follows: inhibitory rate (%) = (1 − T_Wt_/T_Wc_) × 100, where T_Wt_ and T_Wc_ are the mean tumor weight of the treated and control groups, respectively. The inhibitory rates of the co-treatment of ginkgetin–resveratrol with 5-FU were significantly increased dose-dependently when compared with the 5-FU treatment alone (Figure 6E). The treatment of 5-FU plus the combined ginkgetin-resveratrol at high dose also exhibited higher rates of tumor inhibition (~55%) than that of the 5-FU-ginkgetin, or 5-FU-resveratrol group at ~49% or ~47%, respectively. The tumor inhibitory rate in the 5-FU and Avastin co-treated group was ~45% (Supplementary Figures S3E and S5E, Figure 6E).

**Figure 7:**
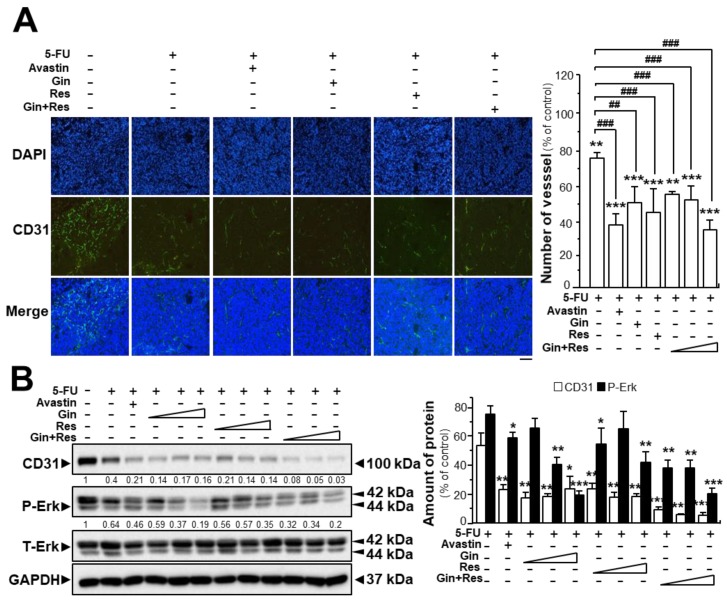
Synergistic activity of ginkgetin with resveratrol reduced microvessel density in tumors. (**A**) Tumors from drug-treated mice were collected, as in Figure 6. Tumors were immune-stained with the anti-CD31 antibody. Green fluorescence represents the antibody staining (left). ImageJ software was used for the quantification of tumor microvessel density (right). (**B**) Expression of CD31 (~100 kDa) and P-Erk (~42 and ~44 kDa) in tumors of drug-treated mice (left). Expression of GAPDH (~37 kDa) served as a control. Quantitation of protein expression is shown (right). Data are expressed as the mean ± SEM of the percentage of change when compared with the control, where *n* = 5; * *p* < 0.05; ** *p* < 0.01; *** *p* < 0.001 vs. the control group; ## *p* < 0.01; ### *p* < 0.001 vs. the 5-FU group. Bar = 100 μm.

The tumors from post-mortem mice were further analyzed. Immunofluorescence staining for the endothelial cell marker CD31 in tumors extracted from mice, was revealed. As expected, the untreated tumor displayed blood vessels with a high vascular density; however, the vascular network of drug-treated groups showed significantly lower vascular density and branches (Supplementary Figures S4 and S6, Figure 7A). The vascular density of tumors in the group co-treated with 5-FU and Avastin was decreased to ~38%, while the vascular density of tumors treated with 5-FU and combined ginkgetin–resveratrol (34.3 ± 2.6%) was lower than that in groups treated with 5-FU and ginkgetin (50.7 ± 14.1%) or resveratrol (44.9 ± 7.1%) (Supplementary Figures S4 and S6, Figure 7A).

Similarly, western blot analysis showed that CD31 expression was lower in tumor tissues of mice treated with 5-FU and ginkgetin–resveratrol than that in the control (Figure 7B). Treatment of 5-FU alone decreased the expression of the CD31 protein to ~45% of the control, and the co-treatment of 5-FU plus Avastin suppressed the expression of the CD31 protein to ~20% of the control. Similarly, the expression of CD31 protein in the group co-treated with 5-FU and the combination of ginkgetin and resveratrol (~4%) was lower than that of the 5-FU–ginkgetin (~19%) or 5-FU–resveratrol group at high dose (~15%) (Figure 7B). Furthermore, the phosphorylation of Erk was significantly decreased in the tumors of mice co-treated with 5-FU and ginkgetin (~20%) or resveratrol (~35%), and the Erk phosphorylation was mostly diminished when mice were administered with 5-FU in combination with ginkgetin–resveratrol (~17%). Co-treatment of 5-FU and Avastin showed similar inhibitory effects on Erk phosphorylation (Figure 7B). These results therefore indicate that ginkgetin and resveratrol suppressed tumor growth in a HT-29 mouse xenograft model by inhibiting angiogenesis within the tumor.

**Figure 8:**
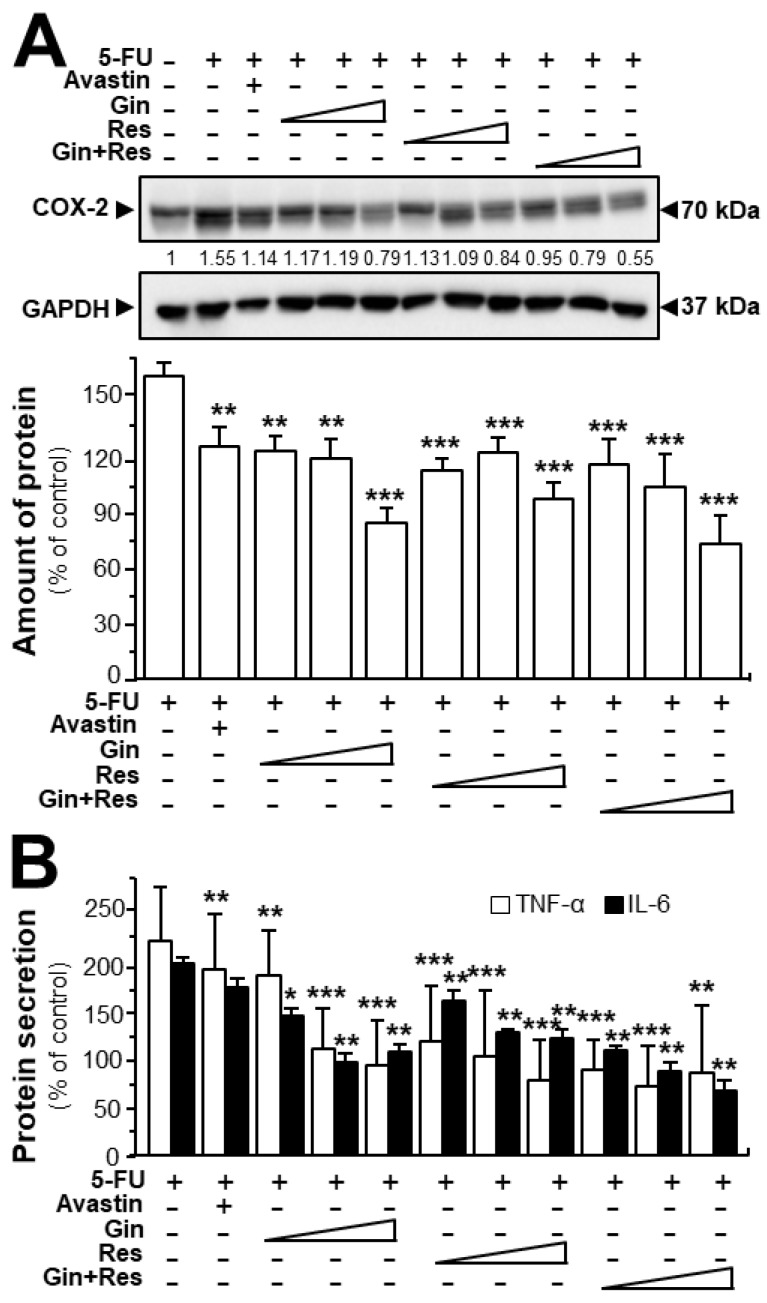
Ginkgetin combined with resveratrol reduced the secretion of inflammatory cytokines in 5-FU-treated mice. (**A**) Expression of COX-2 (~70 kDa) in tumors of drug-treated mice (upper panel). Expression of GAPDH (~37 kDa) served as the control. Quantitation of protein expression is shown (lower panel). (**B**) Blood was collected for cytokine measurement. The expressions of IL-6 and TNF-α in serum were determined by enzyme-linked immunosorbent assay. The drug concentration was in line with Figure 6. Data are expressed as the mean ±SEM of the percentage of change when compared with the control, where *n* = 6; * *p* < 0.05; ** *p* < 0.01; *** *p* < 0.001 vs. the 5-FU group.

In cancer treatment, the continuous use of 5-FU resulted in severe toxicity, particularly intestinal mucositis (e.g., inflammatory responses) [26]. Here, we were interested in determining the effects of ginkgetin–resveratrol in inflammatory mediation. Furthermore, both ginkgetin and resveratrol are well-known safe phytochemicals. As expected, the 5-FU-treated mice showed intense expression of COX-2 when compared with the control group (Figure 8A). However, the treatments of ginkgetin, resveratrol, or combined ginkgetin–resveratrol decreased the 5-FU-induced COX-2 expression in dose-dependent manners (Figure 8A). Moreover, we further examined the expressions of TNF-α and IL-6 by using an ELISA assay. The results showed that the combined ginkgetin–resveratrol treatment significantly reduced the levels of TNF-α and IL-6 cytokines in 5-FU-treated tumor tissues, which were consistent with the expression of COX-2 protein (Figure 8B). The combination of ginkgetin–resveratrol decreased the levels of inflammatory cytokines (i.e., TNF-α and IL-6) in dose-dependent manners, the effects of which showed a decrease of up to 85% and 66%, respectively (Figure 8B). Avastin, the positive control, also demonstrated inhibitory effects on 5-FU-mediated inflammatory responses by decreasing COX-2 expression at ~50% and suppressing TNF-α secretion at ~30% (Figure 8). However, when compared with the 5-FU group, Avastin exerted no significant effects on IL-6 secretion, giving support to the better efficiency of the applied phytochemicals in anti-inflammation treatment. These results indicate that ginkgetin, resveratrol as well as their combination could not only notably increase the anti-tumor efficacy, but also somehow relieve the inflammation-related side-effect of 5-FU.

## 3. Discussion 

Ginkgetin is a major constituent in the leaf of *G. biloba*, a globally used herbal supplement [27]. Different studies on ginkgetin have proposed a series of pharmacological activities [28]. Ginkgetin exerts inhibitory effects on different types of cancers including prostate cancer [29], breast cancer [30], non-small cell lung cancer [31], and colon cancer [32]. Moreover, ginkgetin has been shown to exhibit cytotoxic effects in ovarian and prostate cancers [29,33]. On the other hand, resveratrol, a natural compound commonly found in grapes, peanuts, pines as well as in many Chinese medicinal herbs was demonstrated to exert inhibitory effects on inflammation [34], bladder cancer [35], colon cancer [36], and primary gastric cancer [37]. The dosage used for the treatment of gastric cancer was up to 1500 mg/kg of resveratrol by inducing tumor cell apoptosis. These studies indicate the multi-function and safe usage of resveratrol and ginkgetin.

Here, we provide several lines of evidence to support the possible usage of ginkgetin and resveratrol in cancer therapy, similar to that of the well-known antibody drug, Avastin. Ginkgetin suppressed angiogenesis-associated activities at the cellular level, which obviously inhibited the formation of sub-intestinal vessels in zebrafish embryos in vivo and microvascular sprouting in rat aortic ring ex vivo. In addition, a combination of ginkgetin and resveratrol could produce synergistic effects in suppressing VEGF-mediated angiogenic properties in a concentration-dependent manner. The target of ginkgetin/resveratrol is VEGF. VEGF is regarded as the most important growth factor as well as its receptor VEGFR2 to regulate angiogenesis [38]. Therefore, VEGF and its receptor signaling are popular targets for drug development. The synergistic effects of ginkgetin and resveratrol, as revealed here, could be accounted for by two possibilities: (i) resveratrol binding VEGF may potentiate the binding interactions between ginkgetin and VEGF, and vice versa, thus decreasing the amount of VEGF used for activating VEGFRs; and (ii) ginkgetin and resveratrol bind with VEGF at different binding sites, consequently weakening the interactions of VEGF to VEGFR (i.e., reducing the activation of VEGFR signaling). Here, our study is favorable to the latter possibility.

The blockage of new blood capillary formation reduces the supply of nutrients to tumors, and therefore inhibitor(s) of angiogenesis is able to strengthen the therapeutic effects of any anti-cancer drugs (e.g., 5-FU) [25,39]. Avastin is a recombinant and humanized monoclonal antibody, which is one of the most commonly-used anti-angiogenic drugs for cancer therapy, and works by binding with VEGF. Avastin has been approved for the treatment of non-small cell lung cancer, metastatic colorectal cancer, glioblastoma multiforme as well as the wet age-related macular degeneration [40,41,42]. Inspired by the application of Avastin, we determined the synergy of ginkgetin and resveratrol to increase the efficacy of 5-FU in colon cancer xenograft mice. Therefore, the usage of phytochemicals could be better than that of Avastin, as (i) ginkgetin and resveratrol are available in safe natural products (e.g., ginkgo and grape); (ii) the stability of phytochemicals is better than that of protein; and (iii) the intake of phytochemicals is in pill form, instead of injection.

Resveratrol is a polyphenol commonly found in many natural products, which is able to do the job as that of ginkgetin in VEGF-mediated angiogenesis [21]. Resveratrol has several closely related chemical analogues (i.e., polydatin, pterostilbene, 3,4′,5-trimethoxy-trans-stilbene, and piceatannol), and these analogues are also commonly found in natural products [43]. Indeed, docking and binding analyses have shown their binding to VEGF such as that of resveratrol. The proposed binding sites of these analogues are similar to that of resveratrol. For example, polydatin, isolated from Polygoni Cuspidati Rhizoma et Radix, has been shown to bind VEGF, and which has anti-angiogenesis activities [44]; however, polydatin and resveratrol have no synergy in affecting VEGF functions (date not shown). In light of this scenario, we chose resveratrol, the safest and most commonly used phytochemical, to test for its combination with ginkgetin.

Inflammatory cytokines are able to potentiate tumorigenesis by increasing angiogenic mediators such as VEGF. Amongst these cytokines, IL-1β induced VEGF production in gastric cancer cells through Erk- and p38-dependent pathways [45], and IL-6 dose-dependently induced VEGF release from platelets, further linking the processes of inflammation with angiogenesis [46]. Thus, the inflammatory mediators play key roles in stimulating angiogenesis, mainly through the upregulation of VEGF. The reverse is also true (i.e., VEGF and other pro-angiogenic factors can affect the inflammatory process in several ways). For instance, VEGF is a key molecule in rheumatoid arthritis [47]. An anti-VEGF peptide could decrease the serum level of IL-6 in a collagen-induced arthritis mice model, and alleviate the severity of the disease [47]. Blocking VEGF, thus suppressing angiogenesis, in rheumatoid arthritis can potentially block the nutrient supply to the synovium, inhibit the adhesion and migration of leukocytes, and attenuate the production of cytokines by activated endothelial cells [48]. Both ginkgetin and resveratrol showed anti-inflammatory properties. Ginkgetin reduced arthritic inflammation in rat adjuvant-induced arthritis, while resveratrol showed inhibitory effects on TNF-α-induced IL-1β and MMP-3 production in human rheumatoid arthritis fibroblast-like synoviocytes [49,50]. In contrast, few investigations have supported that Avastin could be used for the treatment of diseases related with inflammation. As being shown here, the anti-inflammatory activities of ginkgetin and resveratrol may be attributed to their suppressive effects on angiogenesis. The combined ginkgetin and resveratrol may synergistically exert anti-inflammatory effects, thus these phytochemicals have the potential of being used in the treatment of inflammation-related diseases such as rheumatoid arthritis.

## 4. Materials and Methods

### 4.1. Reagents and Animals

High-performance liquid chromatography grade acetonitrile (ACN) and formic acid were from Merck (Darmstadt, Germany). Deionized water (18 MΩ cm^−1^) was supplied from a Millipore Milli-Q water system (Milford, MA, USA). Other reagents were of analytical purity. Ginkgetin and resveratrol reference compounds, were bought from Chengdu Institute of Biology (Chengdu, China), and their purities were >98%, detected by high-performance liquid chromatography with diode-array detection. Stock solutions of ginkgetin and resveratrol of 100 mM were freshly prepared in dimethyl sulfoxide (DMSO). Recombinant human VEGF (VEGF165) was obtained from R&D systems (Minneapolis, MN, USA). Dichloro-dihydro-fluorescein diacetate (DCFH-DA) was from Sigma-Aldrich (St. Louis MO, USA). Protein Standards and Ladders were purchased from Thermo Fisher Scientific (Waltham, MA, USA). The following antibodies phospho-eNOS (Ser1177); eNOS; phospho-Akt (Ser473); Akt; phospho-VEGFR2 (Tyr1175) (19A10); VEGFR2 (55B11); phospho-p44/42 MAPK (Erk1/2) (Thr202/Tyr204); p44/42 MAPK (Erk1/2); MMP-2, MMP-9; and CD31 (cluster of differentiation 31) were purchased from Cell Signaling Technology (Danvers, MA). The GAPDH antibody was from Sigma-Aldrich. Zebrafishes, rats, and mice were maintained at the Animal and Plant Care Facility, with approval by the Animal Ethics Committee of HKUST (Cap. 340). The nude mice protocol was approved by Hangzhou Hibio Experimental Animal Ethics Committee (Permit Number: HB1808021) and under the guidelines of the “Principles of Laboratory Animal Care” (NIH publication No. 80-23, revised 1996) and Institutional Animal Care and Use Committees protocol (HBFM3.68-2015.)

### 4.2. Cell Cultures

Human umbilical vein endothelial cells (HUVECs) were obtained from Lonza (San Diego, CA, USA), and the cultures were performed for 3–6 passages. HUVECs were cultured in EGM-2^®^ BulletKit media strictly following the illustrations (Lonza), as described previously [44]. In the cell viability assay, the 3-(4,5-dimethylthiazol-2-yl)-2,5-diphenyltetrazolium bromide (MTT) method was first performed to study the effects of ginkgetin/resveratrol on HUVECs. Briefly, 5 × 10^3^ endothelial cells in 100 μL of medium were seeded on a sterile 96-well plate (Corning Incorporated, New York, NY, USA). After incubation for 24 h, the medium in each well was changed with 100 μL of fresh medium containing VEGF at 10 ng/mL, or a series of concentrations of ginkgetin/resveratrol, as described previously [21]. After drug treatment for 48 h, 10 μL/well of MTT solution (5 mg/mL) was added. After incubating at 37 °C for another 4 h, the medium was aspirated, and 150 μL of 100% dimethyl-sulfoxide (DMSO) was added into each well to dissolve the formazan salt formed. The color intensity of the formazan solution was read under a microplate spectrophotometer, and the wavelength was set at 570 nm. The lactate dehydrogenase (LDH) release was detected with an application of a cytotoxicity detection kit (Roche Diagnostics, Indianapolis, IN, USA). The LDH content of each group was quantified following the below formula: cytotoxicity (%) = (experimental value − low control)/(high control − low control) × 100.

### 4.3. Analyses of Ginkgetin by HPLC

The extract of *G. biloba* leaves was supplied by Yunnan Baiyao Group Tianzihong Pharmaceutical Co. (Kunming, Yunnan, China) and further qualified by following the Pharmacopoeia of the People’s Republic of China (2015). In brief, 50 g dried leaves were smashed into powder and successively dissolved in 1000 mL of 50% ethanol (1:20 w/v) for 72 h as the extraction. Thus, the extraction was a 50% ethanol mixture and the mixture was used for the HerboChips screening. For ginkgetin identification, an Agilent 1200 HPLC series system, equipped with a degasser, an auto-sampler, a binary pump, and a thermos-stated column compartment, was used for the chemical analysis. An Agilent Grace VisionHT C18 column (4.6 × 250 mm, 5 μm) (Agilent Technologies, Santa Clara, CA, USA) was used for chromatographic separation. The details are given in the legend of Supplementary Figure S1.

### 4.4. Molecular Docking

The crystal structure of the protein was obtained from Protein Data Bank (PDB), and the molecules, ginkgetin (PubChem: 5271805), and bevacizumab (PubChem: 24801581) were both obtained from the NCBI-PubChem database. The structures were separately transferred into MOL2 mode for molecular docking analysis by using Chemoffice 2014 (Cambridge Soft, Cambridge, MA, USA). The docking calculation was performed on DockingServer, which was an interface on the basis of websites to cope with all aspects of molecular docking with the application of AutoDock tools. Molecular docking calculations were separately performed based on the bevacizumab/ginkgetin-VEGF protein model. By using the AutoGrid program, affinity (grid) maps of 40 × 40 × 40 Å grid points, corresponding to x, y, and z, and 0.375 Å spacing, were automatically shown, and box center was designed as followed: x: 0.38 Å, y: −2.98 Å and z: 20.51 Å [51]. The binding modes were generated for the most favorable binding interaction, and the energies could be approximately predicted [52]. The binding actions were run with an application of AutoDock and Vina software, and the binding results were shown with PyMOL molecular graphics. 

### 4.5. Immunoprecipitation Assay

The immunoprecipitation assay was performed to confirm the binding interaction between ginkgetin and VEGF. Briefly, 100 µL 0.5 µM ginkgetin solution, with VEGF protein or biotinylated VEGF, was incubated for 1 hour at 4 °C. Then, 100 µL of PureProteome Streptavidin Magnetic Beads were added separately into the VEGF solution and biotinylated VEGF solution. The interaction reaction was taken for 24 h at 4 °C. Then, the tubes containing mixtures were placed into a magnetic stand, allowing the beads to migrate to the magnet. Then, the solution was carefully removed without disturbing the beads. After three rounds of washing with PBS, acetonitrile (ACN) was applied to precipitate the VEGF/biotin-labelled VEGF complex. The supernatant was subjected to UPLC analysis to determine the amount of ginkgetin, as described previously [21].

### 4.6. Surface Plasmon Resonance (SPR)

The binding between ginkgetin and VEGF protein was performed on a Biacore S200 (GE Life Sciences, Pittsburgh, PA, USA) with a GE series dextran-coated (CM5) sensor chip. Briefly, the reaction temperature was set at 25 °C, and HBS-T (150 mM NaCl, 10 mM Hepes, 0.05% polysorbate 20, 3.4 mM EDTA, pH 7.4) was selected as the running buffer. The sensor surface of the chip used for capture was prepared by covalently immobilizing VEGF onto the chip surface according to EDC/NHS (1-Ethyl-3-[3-dimethylaminopropyl] carbodiimide hydrochloride)/N-hydroxysuccinimide) coupling chemistry. After surface activation, the VEGF protein dissolved in coupling buffer (0.1 M acetate buffer, pH 4.5) was put onto the activated surface of the chip until the detected RU (resonance unit) signal of the VEGF protein reached around 6500 RU. To remove the uncoupled protein, the chip with the activated coupled surfaces were washed and reacted with 10 mM glycine–HCl of which the pH was set at 1.5. A series of concentrations of ginkgetin were diluted with running buffer and flown through the chip surface with the VEGF protein coupled. The interaction between the VEGF protein and ginkgetin was detected in real time. A reference channel with no protein immobilized served as the reference control. Data were analyzed with the application of GE Biacore S200 control software (GE Life Sciences, Pittsburgh, PA, USA).

### 4.7. Migration and Tube Formation Assay

A wound-healing assay was performed to determine the cell migration in vitro [21,44]. Briefly, 20 × 10^4^ endothelial cells in 1 mL of medium were seeded on a sterile 12-well plate. After cells were incubated for 24 h, a single and straight wound was made with the removal of attached cells located in the center of the cell monolayer by using a sterile 200 μL plastic pipette tip. After washing with pre-warmed PBS, pictures were taken with the application of a phase-contrast microscope (A_t0_). After drug treatment for 8 h, three wound areas in endothelial cells were determined randomly and pictures taken (A_t8_). Using Tscratch software (CSE lab, Switzerland), the wound area of each well before and after drug treatments was measured; the recovery percentage of HUVECs was quantized based on the following formula: Recovery (%) = A_t0_ − A_t8_/A_t0_ × 100%. In the tube formation assay, Matrigel was used to pre-coat the 12-well plate, and the polymerization s lasted for 1 h at 37 °C, as reported previously [21,53]. Then, HUVECs containing a series of different concentrations of ginkgetin/resveratrol, or 10 ng/mL VEGF, were plated onto each well of plates coated with Matrigel with the cell density set at 20 × 10^4^ cells/well. After incubation for 8 h at 37 °C with the drug, tube-like structures of HUVECs were photographed with a phase-contrast microscope. Images from different drug-treated groups were analyzed by counting the branching points manually, which were located in three fields in each well. The fields were determined randomly.

### 4.8. Zebrafish Angiogenesis

Zebrafish were grown in a system with regular aeration and flow water and maintained at a cycle of 10 h:14 h of dark/light. The temperature was set at 28.5 °C. Healthy embryos were picked, as previously described [21]. Dechlorinated embryos were randomly assigned into different groups and kept in a 12-well plate (8–10 embryos per well). Embryos were incubated with water with or without ginkgetin and VEGF at 28.5 °C. After drug treatment for 48 h, morphological characteristics and eyes of embryos were used to check viability. The embryos were fixed with paraformaldehyde (4%) at 4 °C for at least 20 h, and then the embryos were rinsed separately by PBS with 0.1% Tween 20 solution (PBST) for 5 min, followed by 50% methanol and methanol. The embryos were rinsed continuously with application of PBST solution four times for 5 min each. The embryos were kept in buffer 9.5T for 15 min at room temperature to start with alkaline phosphatase-based vascular staining assay (i.e., immersed in freshly prepared nitro-blue tetrazolium/5-bromo-4-chloro-3-indolyl-phosphate (NBT/BCIP)) (Cell Signaling Technology, Danvers, MA, USA) at room temperature. To avoid interference of NBT/BCIP, the stained embryos were washed independently with PBST three times, each for 5 min. Finally, the images of stained vessels located in sub-intestinal parts of embryos were taken under a stereomicroscope (Nikon AZ100, Nikon Instruments Inc., Melville, NY, USA) equipped with a digital camera (Olympus DP71, Olympus Life Science, Center Valley, PA, USA). ImageJ software (v2.1.4.7, National Institutes of Health, Bethesda, MD, USA) was used to quantify the areas and branches of sub-intestinal vessels.

### 4.9. Aortic Ring Sprouting

The VEGF-induced outgrowth of micro-vessels was determined with an aortic ring sprouting method, with slight modifications [54]. Efforts were made to minimize the suffering and number of animals. Briefly, thoracic aortas were taken out of 6-week-old Sprague-Dawley male rats and washed in pre-warmed PBS. The fibroadipose tissue around the aorta was gently removed by using small scissors under the microscope, and the aortas were carefully cut into 1-mm thick fragments. Then, the fragments were placed into each well of a 24-well plate pre-coated with Matrigel. A total of 100 μL of Matrigel was added to cover the aortic fragments. Matrigel was allowed to polymerize and solidify for 1 h. A total volume of 500 μL endothelial growth medium with or without VEGF and ginkgetin were added into the wells. Aortic fragments were incubated with medium containing 200 μg/mL Avastin, serving as a control. The aortic fragments were observed on day 8 under a phase-contrast microscope. ImageJ software was applied to quantify the microvascular sprouting area.

### 4.10. Western Blot

Western blotting was undertaken to measure the expressions of MMP-2 and MMP-9 as well as the phosphorylations of VEGFR1 (Y103), VEGFR2 (Tyr1175), p44/42 MAPK (Erk1/2), and eNOS (S1177). Before treatment, HUVECs were incubated in medium without serum for 1 h. The cultures, after treatment, were lysed in freshly made low-salt lysis buffer (2% SDS, 10% glycerol, 200 mM 2-mercaptoethanol, 125 mM Tris-HCl, pH 6.8). To measure the expressions of MMP-2 and MMP-9, the cells were rinsed with cold PBS and harvested in a low-salt lysis buffer containing 100 mM HEPES (pH 7.5), 1 mM EGTA, 150 mM NaCl, 1 mM EDTA, 1% NP-40, 1% Triton, 3 mM benzamidine, 1 mM Na_3_VO4, 10 mM NaF, and 20 μg/mL each of aprotinin and leupeptin. The collected cell lysates were vortexed for 10 min, and the insoluble cell debris were removed after centrifugation. The total protein concentrations were measured using the Bradford method, then all lysates were diluted to the same concentration. All protein extracts were separated by using 7% acrylamide gel electrophoresis, and the proteins were transferred onto nitrocellulose membranes at 4 °C [22]. The primary antibody used was at a 1:1000 dilution for 24 h. After rinsing, membranes were incubated with horseradish peroxidase-conjugated secondary anti-rabbit antibody diluted at 1:2000 for 2 h at room temperature. The membrane was rinsed by TBST solution four times for 5 min each. The reactive bands in the membrane were visualized with enhanced chemiluminescence (Invitrogen, Carlsbad, CA, USA). Images of the reactive bands were captured by a Chemidoc Imaging System (Bio-Rad; Hercules, CA, USA). The band intensities of the control group and groups with drug treatment were run on the same piece of gel and pictures taken under standardized ECL conditions were analyzed for the comparison with the application of the related software, which was performed based on a calibration plot from a parallel gel with one of the samples, diluted at a series of different ratios. To quantify the phosphorylation, the band at 10 min from each group was compared with the corresponding control at 0 min (all western blot figures can be found in the Supplementary Figures S7–S13).

### 4.11. Measurement of Reactive Oxygen Species

The formation of ROS was measured with 2′,7′-dichlorofluorescein diacetate (DCF-DA, Sigma, Sigma-Aldrich, St. Louis MO, USA). After incubation with a series of concentrations of ginkgetin/resveratrol and VEGF (10 ng/mL) for 48 h at 37 °C, endothelial cells were reacted with DCF-DA (100 μM) at 37 °C for 30 min. Then, pre-warmed PBS was used to rinse the cells three times for 5 min each to remove the unreacted DCF-DA. Pictures were taken with the application of a laser confocal fluorescent microscopy. Relevant software was used for image analysis, quantifying the total green fluorescence intensity of the photos in each group.

### 4.12. Animal Xenograft

Six-week old male BALB/C nu/nu nude mice with weights of 22–23 g were maintained in pathogen-free conditions at 22 ± 2 °C, at 70% relatively humidity and under a 12-hour light/dark cycle. HT29 cells (5 × 10^4^/0.2 mL PBS) were harvested and subcutaneously implanted into the right flank of nude mice. The tumors were harvested after they reached a size greater than 300 mm^3^ and then were cut into small pieces of approximately equal sizes (Ф = 0.5−1.5 mm). The tumor pieces were transplanted subcutaneously into the right flanks of the nude mice. On day 16 post-tumor implantation, according to the tumor volume of mice, the mice were randomized into 12 groups: the control group, fluorouracil (5-FU, 30 mg/kg/2 days, Sigma-Aldrich), Avastin group (6 mg/kg/2 days), ginkgetin low-dosage group (200 mg/kg/day), ginkgetin middle-dosage group (400 mg/kg/day), ginkgetin high-dose group (800 mg/kg/day), resveratrol low-dosage group (480 mg/kg/day), resveratrol middle-dosage group (960 mg/kg/day), resveratrol high-dose group (1920 mg/kg/day), ginkgetin and resveratrol low-dosage group (80 mg/kg/day + 240 mg/kg/day), ginkgetin and resveratrol middle-dosage group (160 mg/kg/day + 480 mg/kg/day), and ginkgetin and resveratrol high-dose group (320 mg/kg/day + 960 mg/kg/day). Each group consisted of eight mice and all had a similar starting mean tumor volume. 5-FU and Avastin, dissolved in saline, were administered through intraperitoneal injection once in two days. The 5-FU treatment was applied to each group. The ginkgetin solution (2% DMSO, 6% cremophor EL, 92% NaCl) and resveratrol solution (2% DMSO, 98% CMC-Na) were administered intragastrically once per day. Drug treatment lasted for 30 days. Tumor volumes and body weights of mice were measured every other day throughout the treatment period. Tumor volume was measured using a Vernier caliper. The tumor volume (cm^3^) was calculated using the ellipsoid formula: (D × (d^2^))/2, where “D” represents the large diameter of the tumor and “d” represents the small diameter. The mice were sacrificed after 40 days of treatment, and the tumor weights were recorded.

### 4.13. Immunohistochemistry

Fresh mouse tumor tissue was fixed in 4% paraformaldehyde solution and dehydrated in 70% ethanol, 80% ethanol, 90% ethanol, 95% ethanol and 100% ethanol. Tumor tissues were embedded in paraffin for 5 h. The paraformaldehyde/paraffin-embedded tumor samples were then sectioned to 4 μM thicknesses and mounted on microscope slides. De-paraffinized slides used xylene, hydrated through graded alcohol into water. Slides were washed, treated with 3% H_2_O_2_, and blocked for 30 min at 37 °C. Antigen retrieval was performed by heating for 10 min in 0.01 M sodium citrate buffer (pH 6.0). Tissue sections were incubated in primary antibody at 4 °C overnight. After washing in 1× PBS, the samples were incubated with Alexa Fluor 488-conjugated goat anti-rabbit secondary antibody at a 1:500 dilution for 2 h at room temperature in the dark. 4′,6-diamidino-2-phenylindole (DAPI) nuclear staining (5 mg/mL) followed. After washing, tumor sections were observed under light microscope (BX43, Olympus, Japan). Positive areas were quantified by Image-Pro Plus version 6.0 (Media Cybernetics, Media Cybernetics Inc., Rockville, MD, USA).

### 4.14. Measurement of IL-6 and TNF-α

Blood collected from mice was reacted with anti-coagulant, EDTA. Then, the blood mixture was centrifuged at 2000× *g* for 5 min to obtain the serum, stored at −80 °C. Concentrations of interleukin-6 (IL-6) and Tumor Necrosis Factor (TNF)-α were measured by ELISA (Cell Signaling Technology). Microtiter plates were prepared by coating with monoclonal rat anti-IL-6 or anti-TNF-α. Absorbance was determined at 405 nm.

### 4.15. Calculation of Drug-to-Drug Synergism

The multiple drug analysis was to examine drug interaction according to the median-effect principle first described by Chou et al. [55,56]. This involves the plotting of dose effective curves for each drug and two drugs together in different doses using the median effect equation (*F_a_/F_u_ = (D/D_m_*)^m^) where *D* is dose; *D_m_* is the dose required for 50% effect; *F_a_* is the fraction effected by *D*; *F_u_* is the unaffected fraction (1 − *F_a_*); and *m* is the coefficient of sigmoidicity of the dose–effect curve. A combination index (CI) is then determined using the classical isobologram equation of Chou–Talalay: CI = (*D*)_1_/(*D_x_*)_1_ + (D_x_)_2_/(*D_x_*)_2_. (*D_x_*)_1_ is the dose of drug 1 required to produce x% effect alone; (*D*)_1_ is the dose of drug 1 required to produce the same x% effect in combination with (*D*)_2_; (*D_x_*)_2_ is the dose of drug 2 required to produce x% effect alone; and (*D*)_2_ is the dose of drug 2 required to produce the same x% effect in combination with (*D*)_1_. Values of CI referred to CI close to 1 = additive effect; CI > 1 = antagonistic effect; and CI < 1 = synergistic effect. For dose reduction index (DRI), it provides a measure of how much the dose of each drug in a synergistic combination could be reduced at a given effect level (i.e., at x% inhibition) when compared with the doses of each drug alone. The DRI value was calculated as (DRI)_1_ = (*D_x_*)_1_ / (*D*)_1_ and (DRI)_2_ = (*D_x_*)_2_/(*D*)_2_.

### 4.16. Statistics and Others

Statistical analysis was performed by one way analysis of variance (ANOVA), following a Bonferroni multiple comparisons test using the SPSS 16.0 software (V16.0, International Business Machines Corporation, New York, NY, USA). Data were expressed as the mean ± standard error of the mean (SEM) and the statistical significance was set at *p* < 0.05. Protein were measured by the Bradford method with a kit from Bio-Rad (Bio-Rad Laboratories, Inc., Hercules, CA, USA).

## 5. Conclusions

This study illustrates the pharmacological roles of the synergy of ginkgetin and resveratrol in angiogenesis, cancer, and its related signaling mechanisms. The combination of ginkgetin and resveratrol could be used as an angiogenesis inhibitor with potentials for cancer treatment and drug development, which exerts its suppressive effects through a series of steps via interfering with VEGF-related signaling transduction. Such pharmaceutical activities of ginkgetin and resveratrol, unlike other angiogenesis inhibitors, could be further applied for the prevention and treatment of various kinds of diseases that are related with angiogenesis as a novel anti-angiogenesis agent.

## Supplementary Materials

The following are available online at https://www.mdpi.com/2072-6694/11/12/1828/s1, Figure S1: HerboChips screening and identification of ginkgetin. Figure S2: Ginkgetin blocks the VEGF-mediated phosphorylations of VEGFR2, Erk, Akt and eNOS.: Ginkgetin strengthens the cytotoxicity of 5-FU on HT-29 tumour growth in mice. Figure S4: Ginkgetin reduces tumour microvessel density. Figure S5: Resveratrol strengthens the cytotoxicity of 5-FU on HT-29 tumour growth in mice. Figure S6: Resveratrol reduces tumour microvessel density. Figure S7: Supplemental data for Supplementary Figure S2. Figure S8: Supplemental data for Supplementary Figure S2. Figure S9: Supplemental data for Supplementary Figure S2. Figure S10: Supplemental data for Supplementary Figure S2. Figure S11: Supplemental data for Figure 3B. Figure S12: Supplemental data for Figure S7. Figure S13: Supplemental data for Figure 8.

## Abbreviations

HUVECs: human umbilical vein endothelial cells; VEGF, vascular endothelial growth factor; VEGFR1, vascular endothelial growth factor receptor 1; VEGFR2, vascular endothelial growth factor receptor 2; VEGFR3, vascular endothelial growth factor receptor 3; JNK, c-Jun N-terminal kinase; eNOS, endothelial nitric oxide synthase; Akt, protein kinase B; Erk, extracellular signal-regulated kinase; GAPDH, glyceraldehyde 3-phosphate dehydrogenase; MTT, 3-(4,5-dimethylthiazol-2-yl)-2,5-diphenyltetrazolium bromide; DCFH-DA, 2’7’-Dichlorofluorescein diacetate.
