# Supplementary material for: Synergy of Ginkgetin and Resveratrol in Suppressing VEGF-Induced Angiogenesis: A Therapy in Treating Colorectal Cancer
Source: Cancers (Basel). 2019 Nov 20;11(12):1828. doi: 10.3390/cancers11121828 (PMC6966653; doi:10.3390/cancers11121828)
Supplement: Supplementary file 1 [file cancers-11-01828-s001.pdf]

cancers

MDPI

Article

Synergy of Ginkgetin and Resveratrol in Suppressing VEGF-Induced Angiogenesis: A Therapy in Treating Colorectal Cancer

Wei-Hui Hu [1,2], Gallant Kar-Lun Chan [1,2], Ran Duan [1,2], Huai-You Wang [1,2], Xiang-Peng Kong [1,2], Tina Ting-Xia Dong [1,2] and Karl Wah-Keung Tsim [1,2,*]

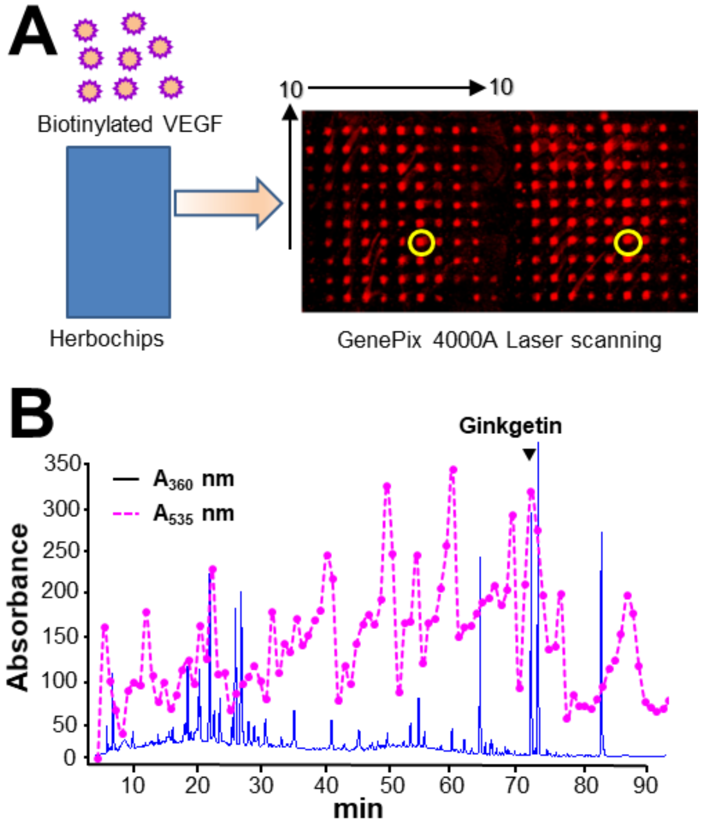

Supplementary Figure S1. HerboChips screening and identification of ginkgetin. (**A**) Fraction (1 min) from herbal extract was gathered by HPLC, and then which was dotted on a chip. The blank chips went through surfaces activation with the application of epoxy groups before dotted with herbal extract. Then, the previously collected herbal fractions were separately dotted on surface of activated chips in rows with application of an automatic arrayer (Biodot A101, Shuai Ran Precision, Taiwan) and then went through fixation. After fixation, the chip was applied to hybridize with biotin-labelled VEGF. After reacting with biotinylated VEGF protein, the chip was used to hybridize with straptavidin-Cy5 and then fluorescence detection was performed under a fluorophore microarray scanner. Representative scanned images obtained from a fluorophore microarray scanner were shown. The protocol about HerboChips screening technology in details could be found in Hu et al., 2018. (**B**) The HPLC separation was performed at room temperature, and the flow rate was set at 0.75 mL/min. The gradient elution applied here was set at followed: Solvent A was increased from 8% to 18% by 12 min, from 18% to 24% by 24 min, from 24% to 25% by 5 min, from 25% to 40% by 15 min and finally increased from 40% to 75% by 96 min. Before sample injection, 0.45 μm Millipore syringe filter unit was used to filter herbal extract. Ten μL of filtered herbal extract and the standard solution of ginkgetin, at a concentration of 1000 mg/L, were separately performed the injection for HPLC

chromatogram analysis; while the wavelength was set at 360 nm, together with a whole spectral scanning from 190 nm to 400 nm. Each fraction was related with 1 min. Ginkgetin was identified at ~72 min. The collected fractions were dotted and fixed on a chip (HerboChips) and biotinylated VEGF was hybridized with the chip. The signal was recognized by SA-Cy5 and scanned at 535 nm for fluorescence scanning.

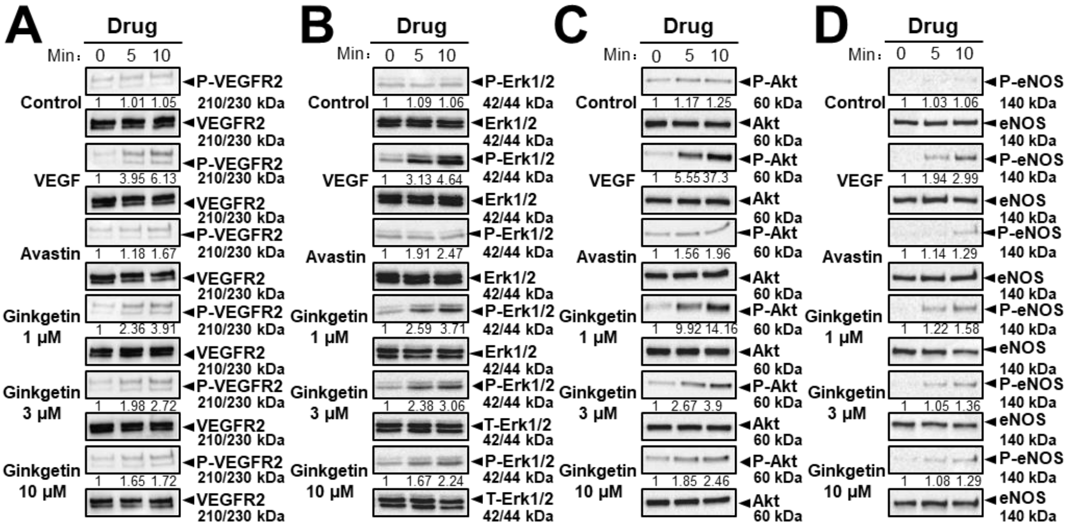

Supplementary Figure S2. Ginkgetin blocks the VEGF-mediated phosphorylations of VEGFR2, Erk, Akt and eNOS. HUVECs were plated into each well of a 12-well plate with the density set at 20 × 10^4 cells per well. The cells were treated with VEGF (10 ng/mL) with or without ginkgetin at demonstrated concentrations. Next cell lysates were collected after 10 min of treatment. Phosphorylated and total protein expressions of **(A)** VEGFR2 at ~210 kDa and ~230 kDa, (**B**) Erk at ~42 kDa and ~44 kDa, (**C**) Akt at ~60 kDa and (**D**) eNOS at ~140 kDa were detected by western blotting. Quantitation was done as shown in Figure 3A. $n = 4$.

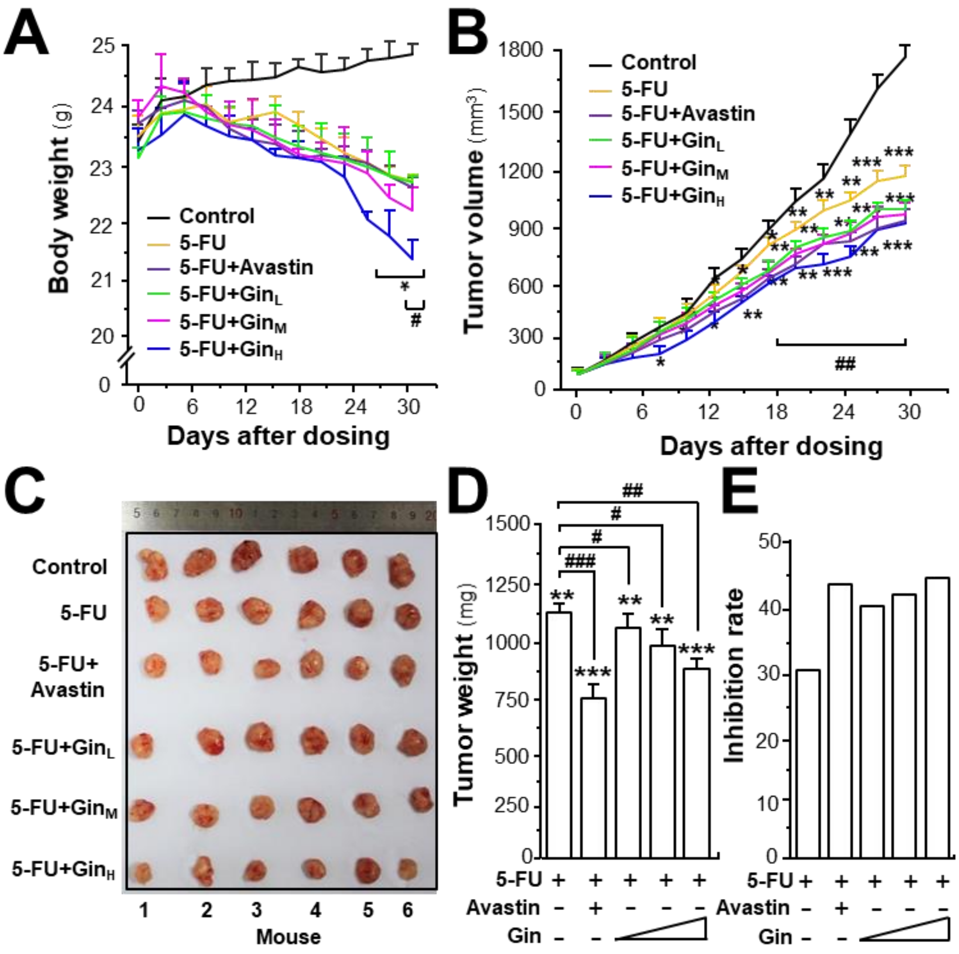

Supplementary Figure S3. Ginkgetin strengthens the cytotoxicity of 5-FU on HT-29 tumour growth in mice. Xenograft nude mice model was established by implanting HT29 colon cancer cells subcutaneously in the right flank of mice. The tumours were allowed to grow at ~90 mm3. Thereafter, 5-FU (30 mg/kg/2 days, i.p.,) Avastin group (6 mg/kg/2 days, i.p.,) ginkgetin low-dosage group (200 mg/kg/day, i.g.), ginkgetin middle-dosage group (400 mg/kg/day, i.g.), ginkgetin high-dose group (800 mg/kg/day, i.g.,) were administered. Mice in each group were administered 5-FU except control group. (**A**) The body weight was measured. (**B**) The mean tumour volume in each group after drug treatment. The tumour volume (in cm^3) was calculated were described as in Fig. 6. (**C**) Mice bearing tumours were sacrificed at day 30, and the tumours were shown. (**D**) Mean tumour weight in each group at the end of treatment. (**E**) Inhibitory rates of drug-treated groups. "5-FU+GinL" referred to the treatment of 5-FU and ginkgetin at low dosage. "5-FU+GinM" referred to the treatment of 5-FU and ginkgetin at middle dosage. "5-FU+GinH" referred to the treatment of 5-FU and ginkgetin at high dosage. Data are expressed as Mean ± SEM of the percentage of change as compared with control, where $n = 8$; * $p < 0.05$;** $p < 0.01$; *** $p < 0.001$ vs. control group; # $p < 0.05$; ## $p < 0.01$; ### $p < 0.001$ vs. 5-FU group.

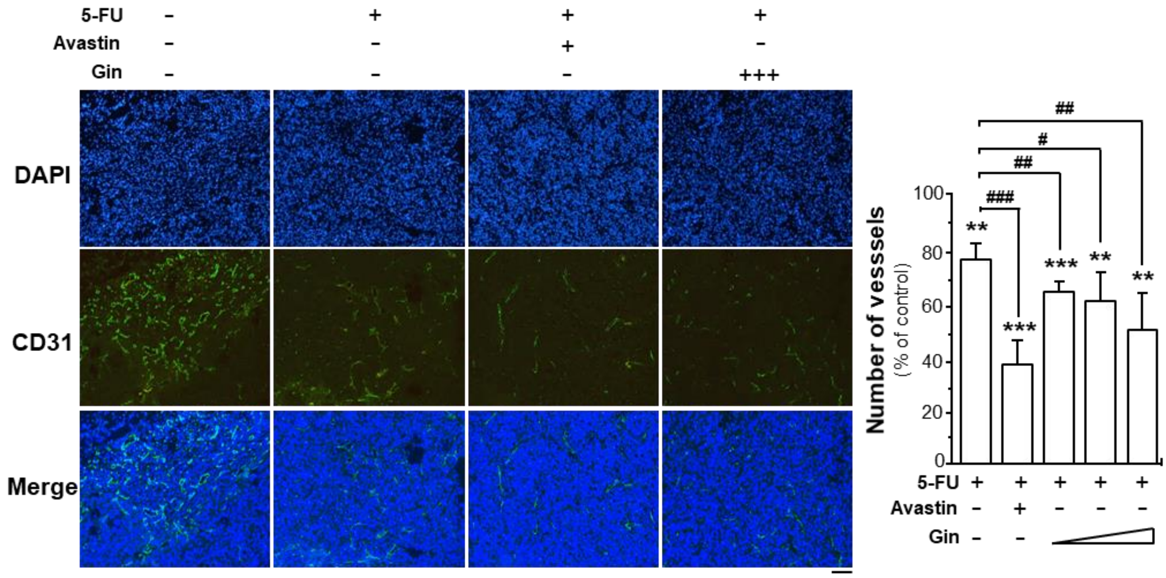

Supplementary Figure S4. Ginkgetin reduces tumour microvessel density. Tumour tissues were immuno-stained with anti-CD31 antibody by performing immunofluorescence staining. Green fluorescence represented the antibody staining (left panel). ImageJ software was used to do the quantification of tumour microvessel density (right panel). Drug concentration was as in Supplementary Figure 3. Data are expressed as Mean ± SEM of the percentage of change as compared with control, where $n = 4$; * $p < 0.05$; ** $p < 0.0$; *** $p < 0.001$ vs. control group; # $p < 0.05$; ## $p < 0.01$; ### $p < 0.001$ vs. 5-FU group. Bar = 100 μm.

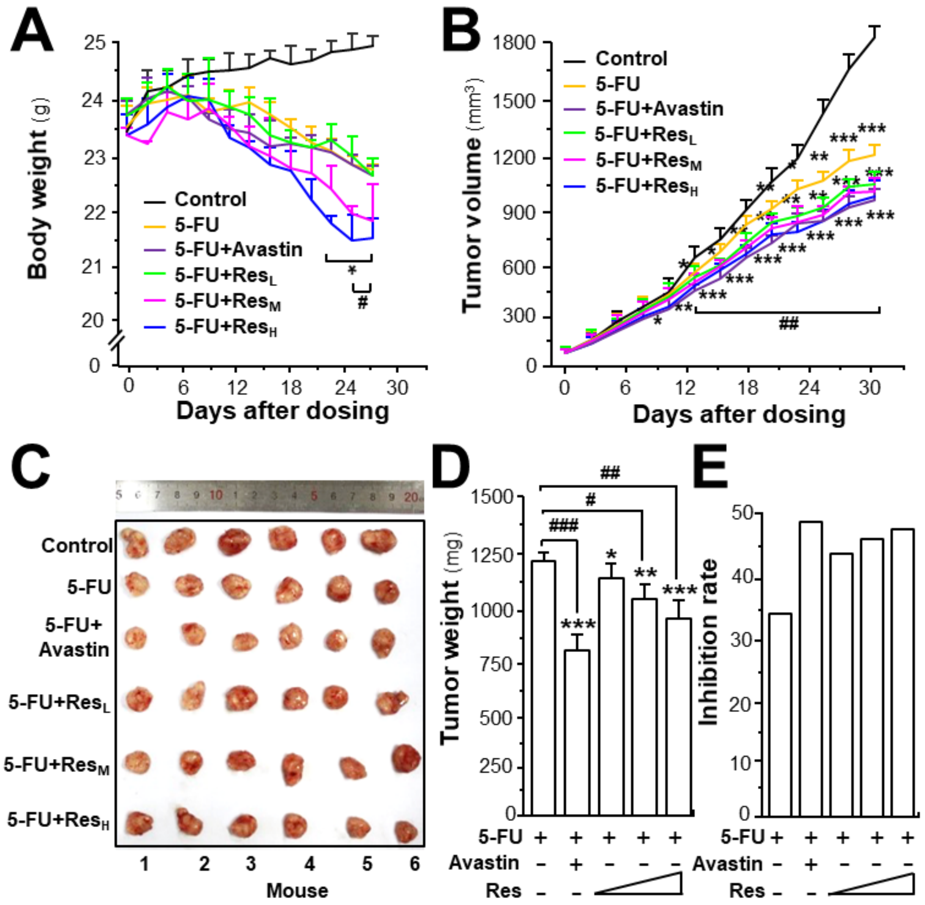

Supplementary Figure S5. Resveratrol strengthens the cytotoxicity of 5-FU on HT-29 tumour growth in mice. Xenograft nude mice model was established by implanting HT29 colon cancer cells

subcutaneously in the right flank of mice. The tumours were allowed to grow at ~90 mm3. Thereafter, 5-FU (30 mg/kg/2 days, i.p.), Avastin group (6 mg/kg/2 days, i.p.), resveratrol low-dosage group (480 mg/kg/day, i.g.), resveratrol middle-dosage group (960 mg/kg/day, ig), resveratrol high-dose group (1,920 mg/kg/day, i.g.) were administered. Mice in each group were administered 5-FU except control group. (**A**) The body weight was measured. (**B**) The mean tumour volume in each group after drug treatment. The tumour volume (in cm^3) was calculated were described as in Fig. 6. (**C**) Mice bearing tumours were sacrificed at day 30, and the tumours were shown. (**D**) Mean tumour weight in each group at the end of treatment. (**E**) Inhibitory rates of drug-treated groups. "5-FU+ResL" referred to the treatment of 5-FU and resveratrol at low dosage. "5-FU+ResM" referred to the treatment of 5-FU and resveratrol at middle dosage. "5-FU+ResH" referred to the treatment of 5-FU and resveratrol at high dosage. Data are expressed as Mean ± SEM of the percentage of change as compared with control, where $n = 8$; * $p < 0.05$; ** $p < 0.01$; *** $p < 0.001$ vs. control group; # $p < 0.05$; ## $p < 0.01$; ### $p < 0.001$ vs. 5-FU group.

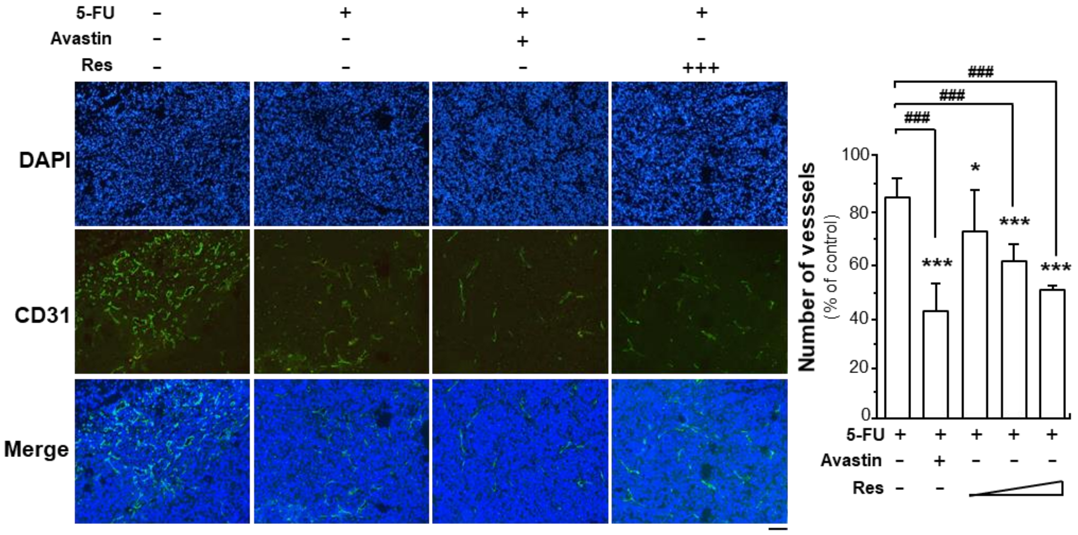

Supplementary Figure S6. Resveratrol reduces tumour microvessel density. Tumour tissues were immuno-stained with anti-CD31 antibody by performing immunofluorescence staining. Green fluorescence represented the antibody staining (left panel). ImageJ software was used to do the quantification of tumour microvessel density (right panel). Drug concentration was as in Supplementary Figure 5. Data are expressed as Mean ± SEM of the percentage of change as compared with control, where $n = 4$; * $p < 0.05$; *$p < 0.01$; *** $p < 0.001$ vs. control group; # $p < 0.05$; ## $p < 0.01$; ### $p < 0.001$ vs. 5-FU group. Bar = 100 μm.

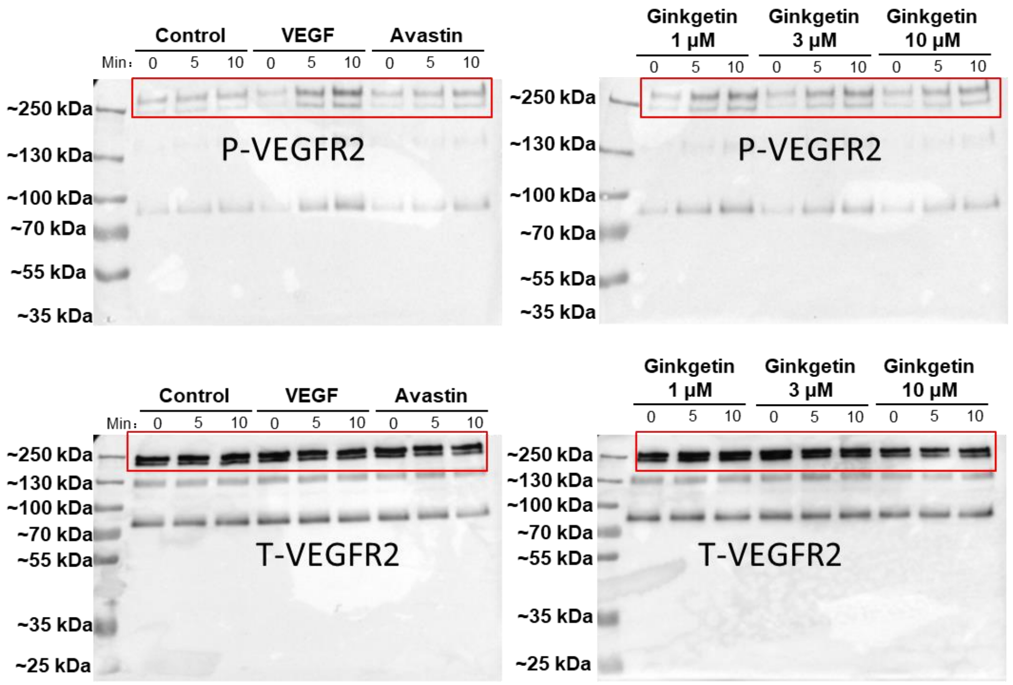

Supplementary Figure S7. Supplemental data for Supplementary Figure S2. Effects on expressions of P-VEGFR2 and T-VEGFR2. Representative unprocessed western blots of control and drugs treated groups as shown in Supplementary Figure S2. Red box showed cropped area included in Supplementary Figure S2.

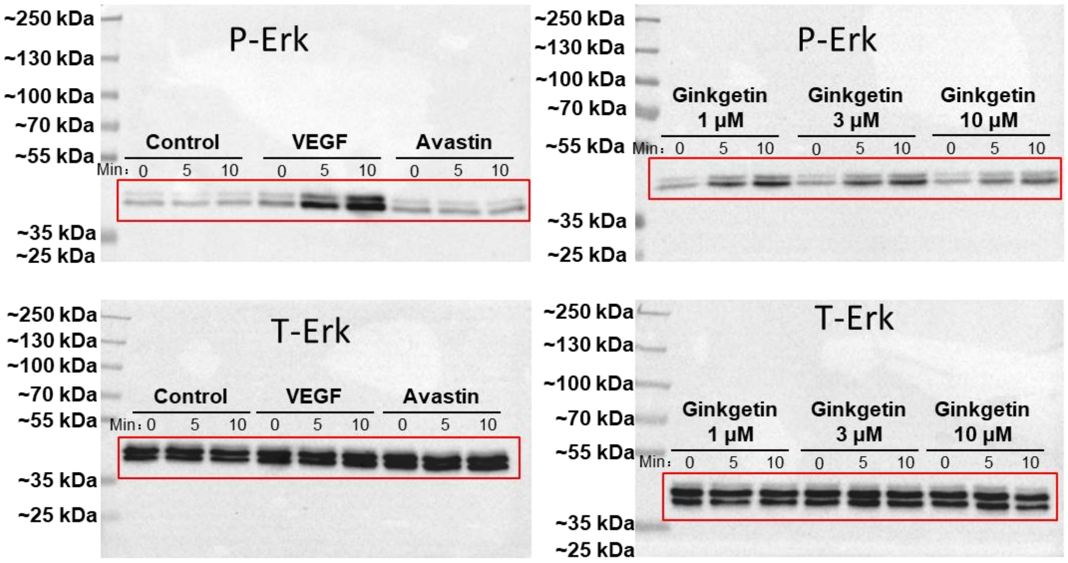

Supplementary Figure S8. Supplemental data for Supplementary Figure S2. Effects on expressions of P-Erk and T-Erk. Representative unprocessed western blots of control and drugs treated groups as shown in Supplementary Figure S2. Red box showed cropped area included in Supplementary Figure S2.

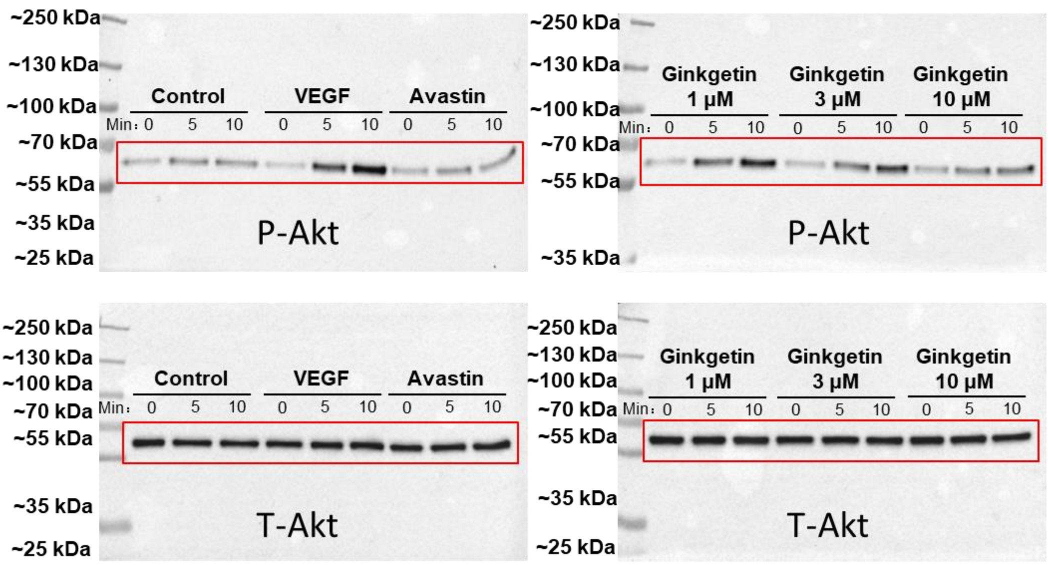

Supplementary Figure S9. Supplemental data for Supplementary Figure S2. Effects on expressions of P-Akt and T-Akt. Representative unprocessed western blots of control and drugs treated groups as shown in Supplementary Figure S2. Red box showed cropped area included in Supplementary Figure S2.

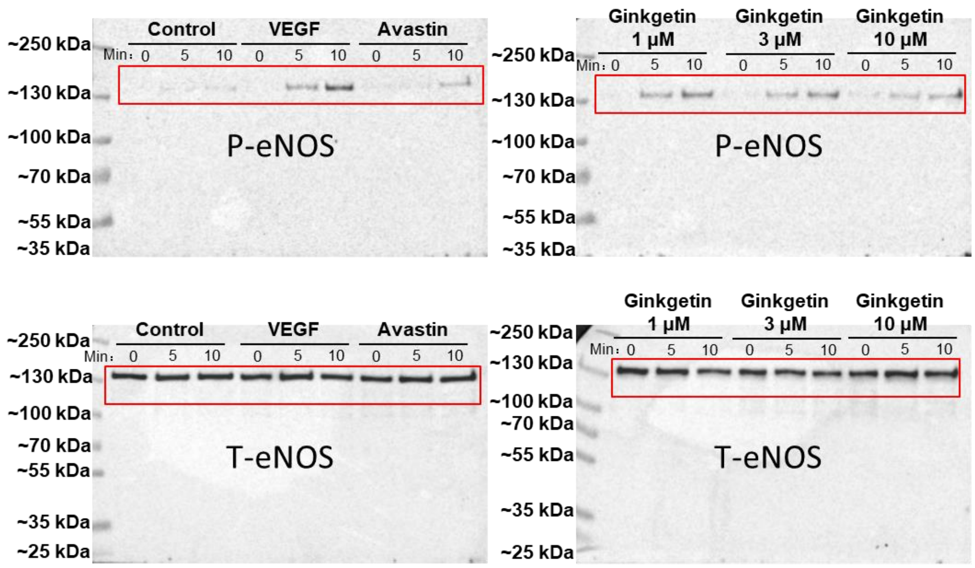

Supplementary Figure S10. Supplemental data for Supplementary Figure S2. Effects on expressions of P-eNOS and T-eNOS. Representative unprocessed western blots of control and drugs treated groups as shown in Supplementary Figure S2. Red box showed cropped area included in Supplementary Figure S2.

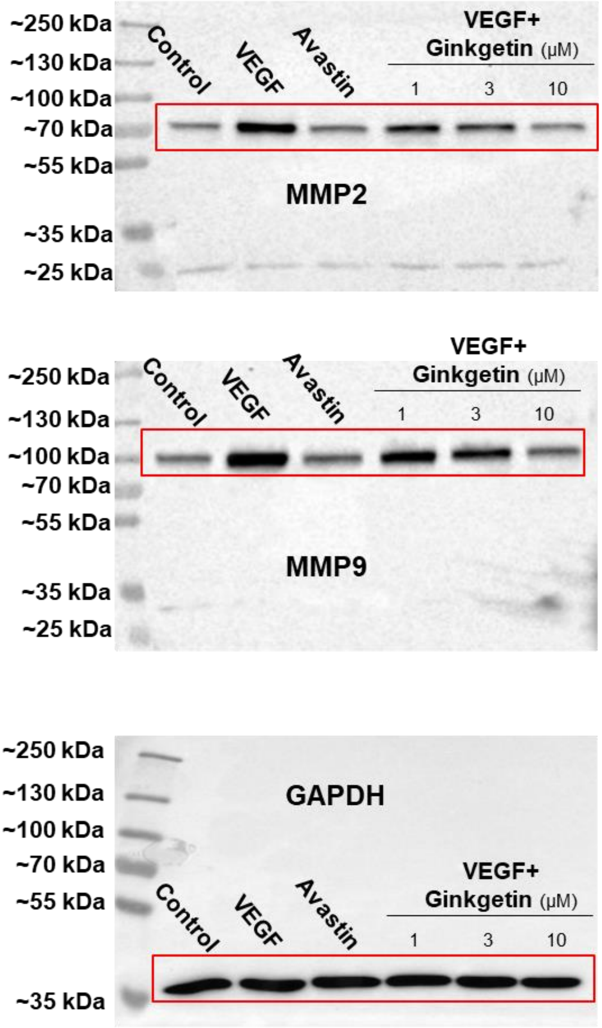

Supplementary Figure S11. Supplemental data for Figure 3B. Effects on expressions of MMP2, MMP9 and GAPDH. Representative unprocessed western blots of control and drugs treated groups as shown in Figure 3B. Red box showed cropped area included in Figure 3B.

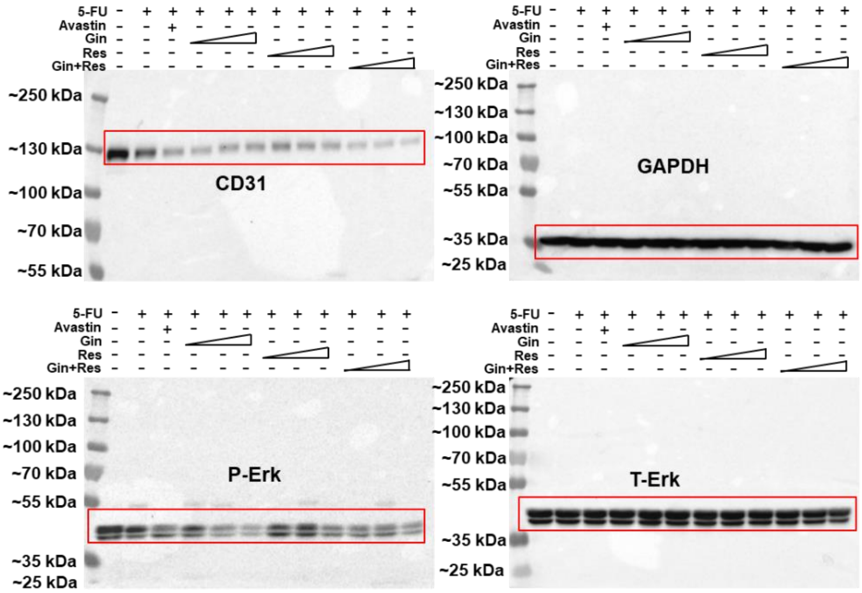

Supplementary Figure S12. Supplemental data for Figure S7. Effects on expressions of CD31, GAPDH, P-Er and T-Erk. Representative unprocessed western blots of control and drugs treated animal tumors as shown in Figure 7. Red box showed cropped area included in Figure 7.

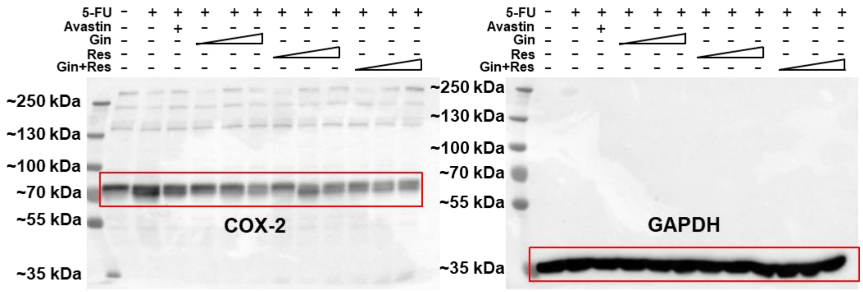

Supplementary Figure S13. Supplemental data for Figure 8. Effects on expressions of COX-2 and GAPDH. Representative unprocessed western blots of control and drugs treated animal tumors as shown in Figure 8. Red box showed cropped area included in Figure 8.

© 2019 by the authors. Submitted for possible open access publication under the terms and conditions of the Creative Commons Attribution (CC BY) license (http://creativecommons.org/licenses/by/4.0/).
